# Ror2 signaling regulates Golgi structure and transport through IFT20 for tumor invasiveness

**DOI:** 10.1038/s41598-016-0028-x

**Published:** 2017-01-26

**Authors:** Michiru Nishita, Seung-Yeol Park, Tadashi Nishio, Koki Kamizaki, ZhiChao Wang, Kota Tamada, Toru Takumi, Ryuju Hashimoto, Hiroki Otani, Gregory J. Pazour, Victor W. Hsu, Yasuhiro Minami

**Affiliations:** 1 Division of Cell Physiology, Department of Physiology and Cell Biology, Kobe University, Graduate School of Medicine, Kobe, 650-0017 Japan; 2 Division of Rheumatology, Immunology and Allergy, Brigham and Women’s Hospital, and Department of Medicine, Harvard Medical School, Boston, MA 02115 USA; 3 RIKEN Brain Science Institute, Wako, 351-0198 Japan; 4 Department of Developmental Biology, Faculty of Medicine, Shimane University, Izumo, 690-8504 Japan; 5 Program in Molecular Medicine, University of Massachusetts Medical School, Worcester, MA 01605 USA

## Abstract

Signaling through the Ror2 receptor tyrosine kinase promotes invadopodia formation for tumor invasion. Here, we identify intraflagellar transport 20 (IFT20) as a new target of this signaling in tumors that lack primary cilia, and find that IFT20 mediates the ability of Ror2 signaling to induce the invasiveness of these tumors. We also find that IFT20 regulates the nucleation of Golgi-derived microtubules by affecting the GM130-AKAP450 complex, which promotes Golgi ribbon formation in achieving polarized secretion for cell migration and invasion. Furthermore, IFT20 promotes the efficiency of transport through the Golgi complex. These findings shed new insights into how Ror2 signaling promotes tumor invasiveness, and also advance the understanding of how Golgi structure and transport can be regulated.

## Introduction

Ror2 is a member of the Ror-family of receptor tyrosine kinases, acting as a receptor for Wnt5a^1^. Wnt5a/Ror2 signaling activates primarily the ß-catenin-independent non-canonical Wnt pathways, which involve various signal mediators, such as Dishevelled, c-Jun N-terminal kinase (JNK), filamin A, c-Src, and Ca^2+^, thereby regulating planar cell polarity and polarized cell motility^1–9^. Wnt5a/Ror2 signaling has also been shown to inhibit the ß-catenin-dependent pathway^10^. Under physiological conditions, the expression of Wnt5a and Ror2 is regulated, leading to modulated Ror2 signaling, such as that seen in development^11–13^. In contrast, higher expression levels of Wnt5a and Ror2 are often seen in various tumor types, resulting in the constitutive activation of Ror2 signaling, which occurs in a cell-autonomous manner^14, 15^.

In this setting, we have previously shown that the expression of both Wnt5a and Ror2 is dependent, at least in part, on the epithelial-to-mesenchymal transition (EMT)-related transcription factor Snail in human osteosarcoma SaOS2 cells^16^. Wnt5a/Ror2 signaling then activates the transcription factor AP-1, which in turn induces the expression of the matrix metalloproteinase (MMP)-13 ^4, 6^. MMP-13 becomes secreted to the extracellular environment, where it degrades the extracellular matrix (ECM) to promote tumor invasion^4^. In addition to MMP-13, other MMPs, such as MMP-2 and membrane type 1-MMP (MT1-MMP), also promote tumor invasiveness^17^. MMPs are targeted to discrete structures on the surface of tumor cells, known as invadopodia, which provide a way of concentrating and targeting MMPs to specific sites of the ECM in promoting tumor invasion^18, 19^.

To achieve these properties of tumor invasion, the intracellular transport of proteins and membranes to the cell surface must be polarized. The Golgi complex has been found to play a key role in promoting this polarization, which requires the Golgi to adopt a ribbon-like structure^20–22^. Early studies showed that the disruption of microtubules (MTs), such as treating cells with nocodazole (NZ), disperses Golgi ribbons into mini-stacks^23, 24^. More recently, new insights into the nature of the MT network that promotes Golgi ribbon formation have emerged. In contrast to the traditional organization of the MT network, which emanates from the centrosome, the MT network that promotes Golgi ribbon formation emanates from the Golgi^25, 26^. Nucleation of Golgi-derived MTs can be promoted through CLASPs (CLIP-associated proteins) interacting with GCC185, which occurs on the *trans*-side of the Golgi^25, 26^, or AKAP450 interacting with GM130, which occurs on the *cis*-side of the Golgi^27^.

MTs also underlie the formation of the cilium, which is a sensory organelle that protrudes from the cell surface and plays important roles in tissue homeostasis and development^28, 29^. Components of the cilium are transported along ciliary MTs by intraflagellar transport (IFT), a conserved motility process^30^. Protein complexes, IFT-A and IFT-B, composed of ∼20 different IFT proteins, serve as cargo adaptors to transport proteins bidirectionally between the base and tip of the cilium. Among the IFT proteins, only IFT20, a component of the IFT-B, has been shown to localize at the Golgi apparatus in addition to the basal body and cilia^31^. A Golgi-localized protein, known as GMAP210, anchors IFT20 to the Golgi membrane, where IFT20 is believed to function in the sorting and/or transport of proteins to the cilium^31–33^. Notably, multiple tumor types lose their cilium during transformation, but IFT20 is still expressed in these non-ciliated tumor cells. What role it may play remains unclear.

Non-ciliary roles for IFT20 are being elucidated in other circumstances. In T cells, which are non-ciliated cells, IFT20 has been detected at the early endosomes, the Golgi and the centrosome^34^. By associating with Rab5 and the T-cell receptor (TCR) at the early endosomes, IFT20 has been found to promote polarized endocytic recycling of the TCR to the immune synapse, which is essential for T cell activation^34–36^. In osteoblasts, IFT20 has been suggested to promote ER-to-Golgi transport of type I collagen, but how it acts in this manner remains to be defined^37^. Here, we uncover a new non-ciliary role of IFT20, acting to regulate Golgi structure and transport, and also find that this role mediates the ability of constitutively activated Ror2 signaling to promote tumor invasiveness. We also elucidate how IFT20 achieves these roles.

## Results

### Ror2 signaling induces IFT20 expression to promote tumor invasiveness

**Figure 1:**
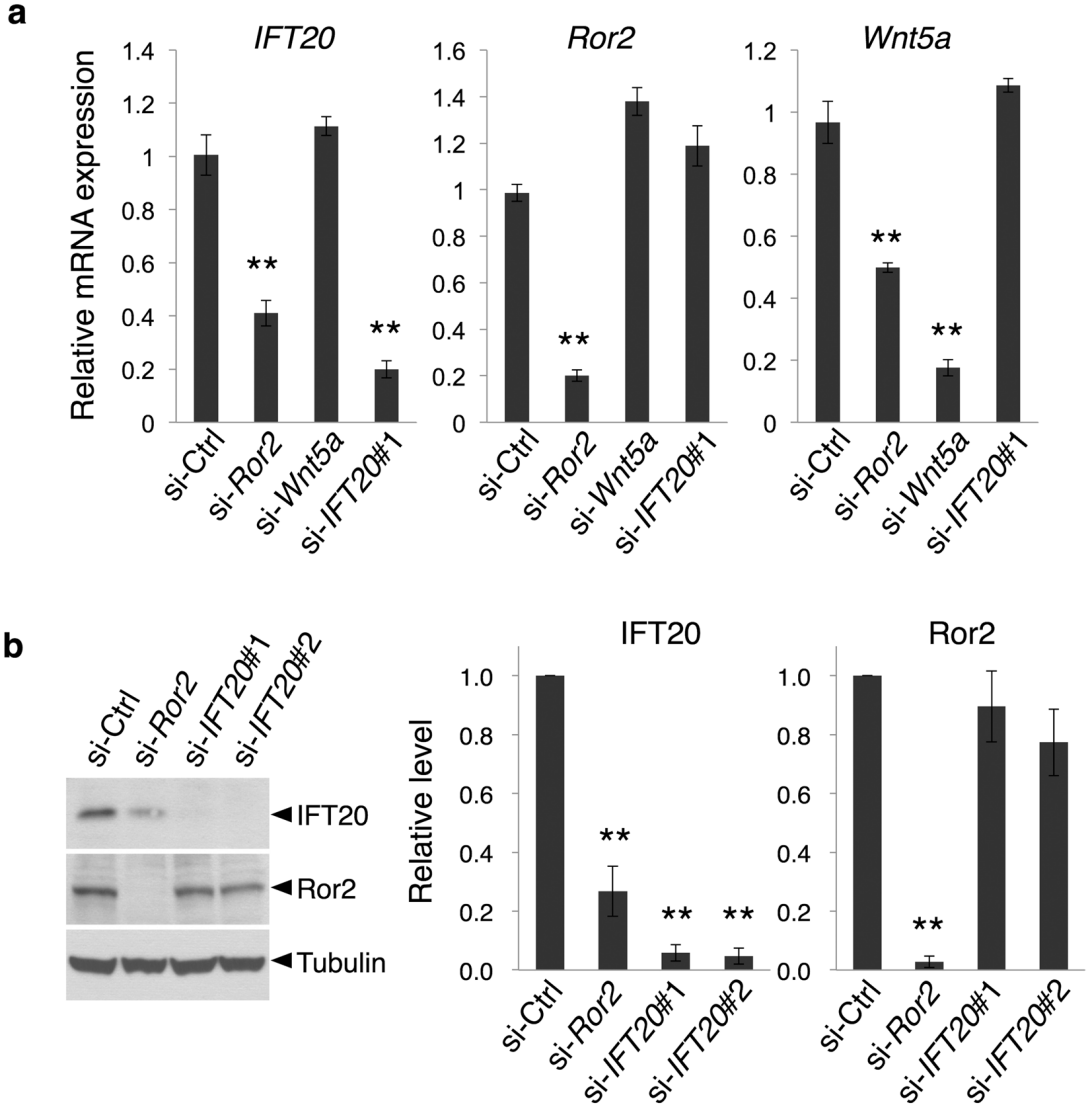
Expression of IFT20 is down-regulated following suppressed expression of Ror2 in SaOS2 cells. (**a**) Quantitative RT-PCR analysis showing decreased expression levels of *IFT20* in si-*Ror2*-transfected SaOS2 cells. Data are expressed as mean ± SD of four independent experiments (**P < 0.005, *t* test). (**b**) Western blot analysis showing decreased protein levels of IFT20 in SaOS2 cells transfected with either *Ror2* or *IFT20* siRNA. Whole cell lysates from the respective cells were analyzed by Western blotting with antibodies against the indicated proteins. The histograms indicate the relative levels of IFT20 and Ror2. Data are expressed as mean ± SD of four independent experiments (**P < 0.005, *t* test).

To gain new insight into how constitutively activated Ror2 signaling promotes tumor invasiveness, we initially performed a microarray analysis on a human osteosarcoma cell line, SaOS2 (GEO accession number: GSE76535). The expression level of *IFT20* mRNA was found to decrease to ∼40% in cells treated with siRNAs for *Ror2*, as compared with those in control cells. These findings were confirmed by quantitative RT-PCR (Fig. 1a) and Western blotting (Fig. 1b). We have also found that siRNA against *Wnt5a* did not affect *IFT20* expression (Fig. 1a), suggesting that IFT20, induced by Ror2 signaling, is likely to be independent of Wnt5a.

Confocal microscopy using antibodies against acetylated-tubulin and Arl13B, which track cilium formation^38, 39^, revealed that SaOS2 cells are non-ciliated (Supplementary Fig. 1). As control, the same culture condition led to cilium formation in human bone marrow-derived mesenchymal stem cells (hMSCs) (Supplementary Fig. 1). Thus, we next examined whether, and how, IFT20 could have a cilium-independent function in SaOS2 cells.

**Figure 2:**
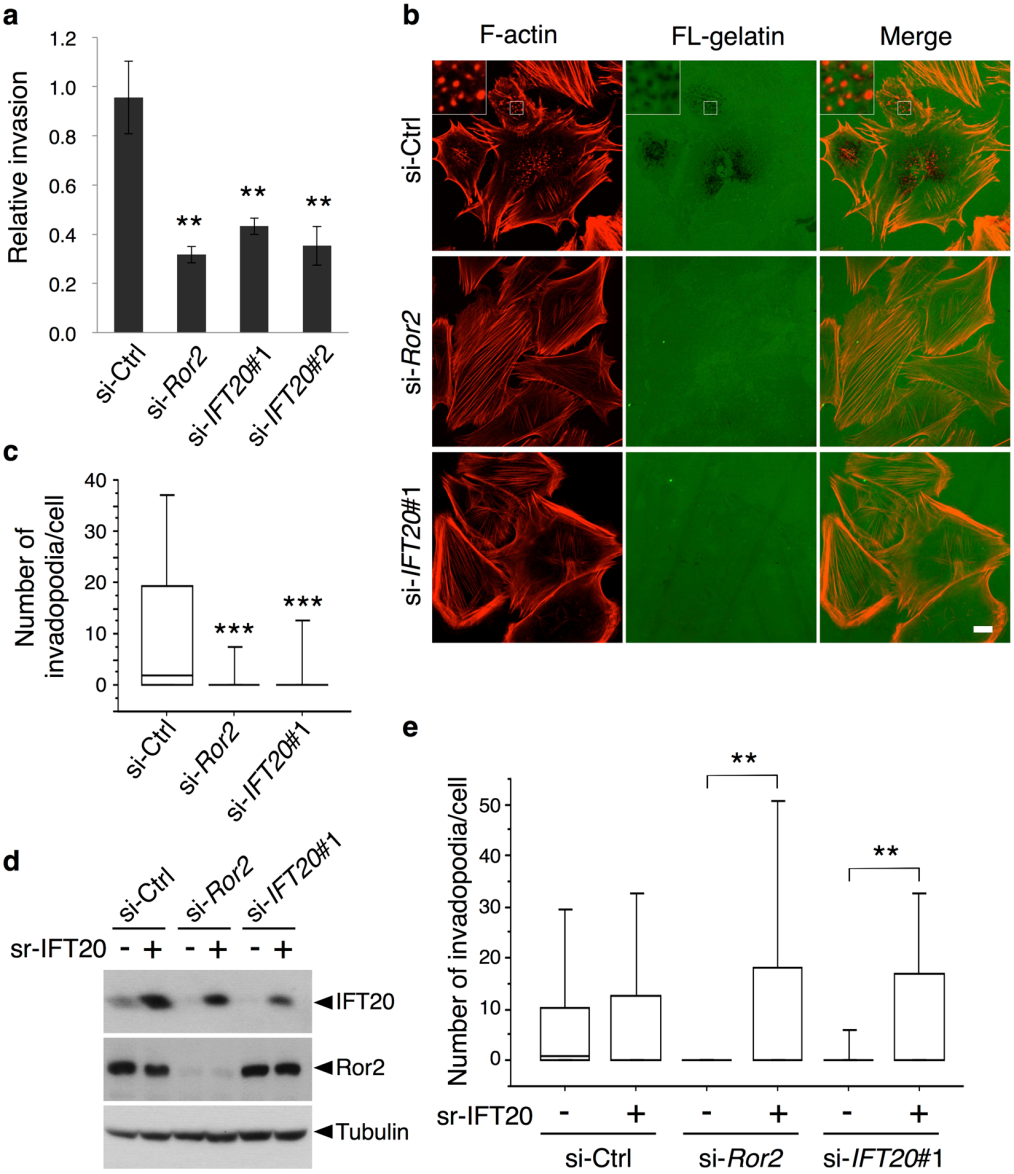
IFT20 plays important roles in invadopodia formation. (**a**) Suppressed expression of *Ror2* or *IFT20* inhibits invasive migration of SaOS2 cells. SaOS2 cells were transfected with the indicated siRNAs and analyzed by Transwell invasion assay. Cells invaded to the lower surface of the Transwell membranes were counted. Data are expressed as mean ± SD of three independent experiments (**P < 0.01, *t* test). (**b**) SaOS2 cells transfected with the indicated siRNAs were cultured on glass coverslips pre-coated with fluorescein-conjugated gelatin (FL-gelatin) for 6 hr and stained with rhodamine-conjugated phalloidin for F-actin. Insets show magnified images of boxed regions. Note that suppressed expression of *Ror2* or *IFT20* inhibits dot-like accumulation of F-actin and degradation of the FL-gelatin, signs of invadopodia formation. Scale bar, 10 µm. (**c**) Quantification of the data shown in (**b**). Number of invadopodia, identified as F-actin dots in the areas of degraded FL-gelatin, per cell was counted. Data are presented as a box-and-whisker plot. n = 127–178, three independent experiments; ***P < 0.001, *t* test. (**d,e**) Reduced activity of invadopodia formation by suppressed expression of *Ror2* or *IFT20* can be rescued by ectopic expression of IFT20. SaOS2 cells transfected with si-Ctrl, si-*Ror2* or si-*IFT20*#1 were further transfected with *pIRES2-ZsGreen1-siRNA-resistant* (*sr*)-*IFT20* (+) or *pIRES2-ZsGreen1* (−), as indicated. The respective transfected cells were subjected to Western blotting with antibodies against the indicated proteins (**d**) or plated on glass coverslips pre-coated with Alexa Fluor 596-conjugated gelatin (Alexa-gelatin) (**e**). Invadopodia formation of ZsGreen1-positive cells were assessed and quantified as in (**c**). Data are presented as a box-and-whisker plot. n = 40–44, three independent experiments; **P < 0.01, *t* test.

Because Ror2-mediated signaling promotes the invasiveness of these tumor cells^4^, we initially explored whether IFT20 could have a role in this process. A transwell invasion assay revealed that suppressing the expression of either *Ror2* or *IFT20* inhibited invasive cell migration through Matrigel (Fig. 2a). As tumor invasion involves invadopodia formation, and we have previously shown that Ror2-mediated signaling promotes invadopodia formation in SaOS2 cells^4^, we next examined whether IFT20 is required for invadopodia formation. Cells were cultured on glass cover slips pre-coated with fluorescein-labeled gelatin (FL-gelatin). Invadopodia formation was assessed by monitoring the F-actin dots in the areas of degraded FL-gelatin, which revealed that siRNA against either *Ror2* or *IFT20* led to significant inhibition (Fig. 2b,c). Notably, the ectopic expression of siRNA-resistant (sr)-IFT20 reverted not only the effect of siRNA against IFT20, which confirms the specificity of the siRNA targeting, but also the effect of siRNA against Ror2 (Fig. 2d,e). This latter finding revealed that Ror2 signaling acts through IFT20 to promote invadopodia formation.

### IFT20 regulates Golgi ribbon structure

**Figure 3:**
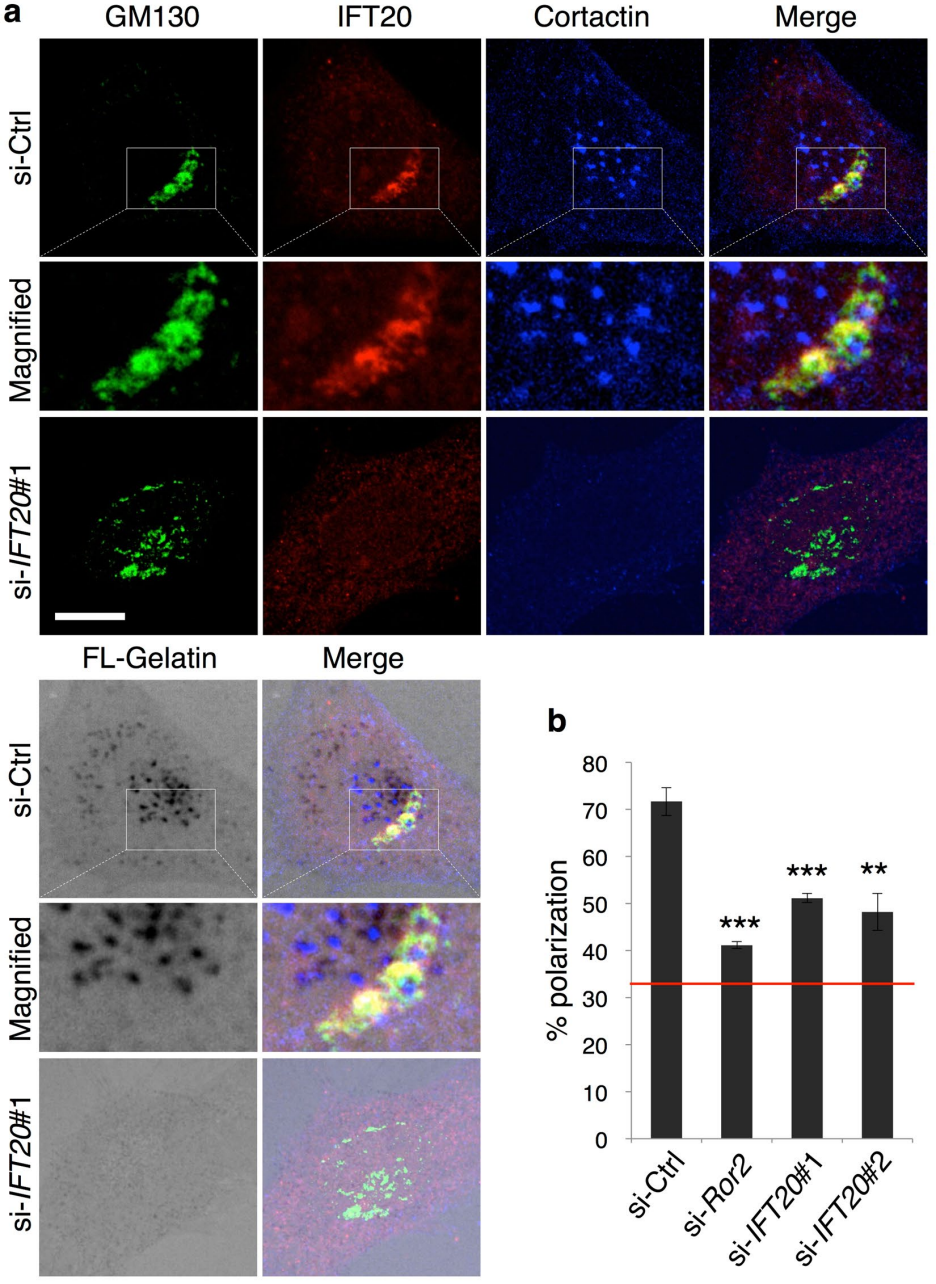
IFT20 is required for reorientation of the centrosome toward the direction of cell invasion. (**a**) Localization of IFT20 at the *cis*-Golgi in SaOS2 cells. Cells transfected with si-Ctrl or si-*IFT20*#1 were cultured on FL-gelatin-coated glass coverslips for 6 hr. Cells were then stained with antibodies against IFT20 and cortactin, and counterstained with an antibody, which is conjugated to Alexa Fluor 647 and directed against GM130, a *cis*-Golgi marker. Boxed regions are magnified on the middle of each image. Note that invadopodia, indicated by a dot-like accumulation of cortactin in the areas of degraded FL-gelatin, are formed in the close vicinity to the Golgi apparatus, where IFT20 is localized. Scale bar, 10 µm. (**b**) SaOS2 cells transfected with the indicated siRNAs were examined for reorientation of the centrosome using 2D invasion assay (see Methods). The percentages of the edge cells in which the centrosome were detected within the 120° sector emerging from the center of the nucleus and facing toward the space were measured. The red line in the graph indicates the level of expected random orientation of 33%. Data are expressed as mean ± SD of three independent experiments (**P < 0.01, ***P < 0.001, *t* test).

To gain insight into how IFT20 acts in this manner, we next assessed the intracellular distribution of IFT20 in SaOS2 cells. Confocal microscopy revealed that a significant pool of IFT20 exists at the Golgi (Fig. 3a), in particular at the *cis*-side of this organelle, as reflected by IFT20 colocalizing better with GM130 (a *cis*-Golgi marker) than with Golgin-97 (a *trans*-Golgi marker) (Supplementary Fig. 2). Moreover, the Golgi was positioned in close proximity to invadopodia structures, which could be identified as dots-like accumulation of cortactin, a marker of invadopodia, in the areas of degraded FL-gelatin in control cells but not *IFT20* siRNA-treated cells (Fig. 3a).

During tumor cell invasion, the Golgi and centrosome have been found to reorient toward the direction of invasion in providing polarized delivery of secretory proteins, such as MMPs, to invadopodia^40^. Thus, we next examined whether IFT20 is involved in this polarization during tumor cell invasion. Because siRNA against IFT20 disrupts the ribbon morphology of the Golgi (Fig. 3a), we performed a 2D invasion assay, and assessed polarization by tracking the positioning of the centrosome (see the Methods section for details). In the control condition, we found that 70% of the cells exhibited the polarization of their centrosome, which was about 2 fold over that expected for random orientation (33%) (Fig. 3b). In contrast, siRNA against either *Ror2* or *IFT20* reduced the polarization of cells to about 40% and 50%, respectively (Fig. 3b).

**Figure 4:**
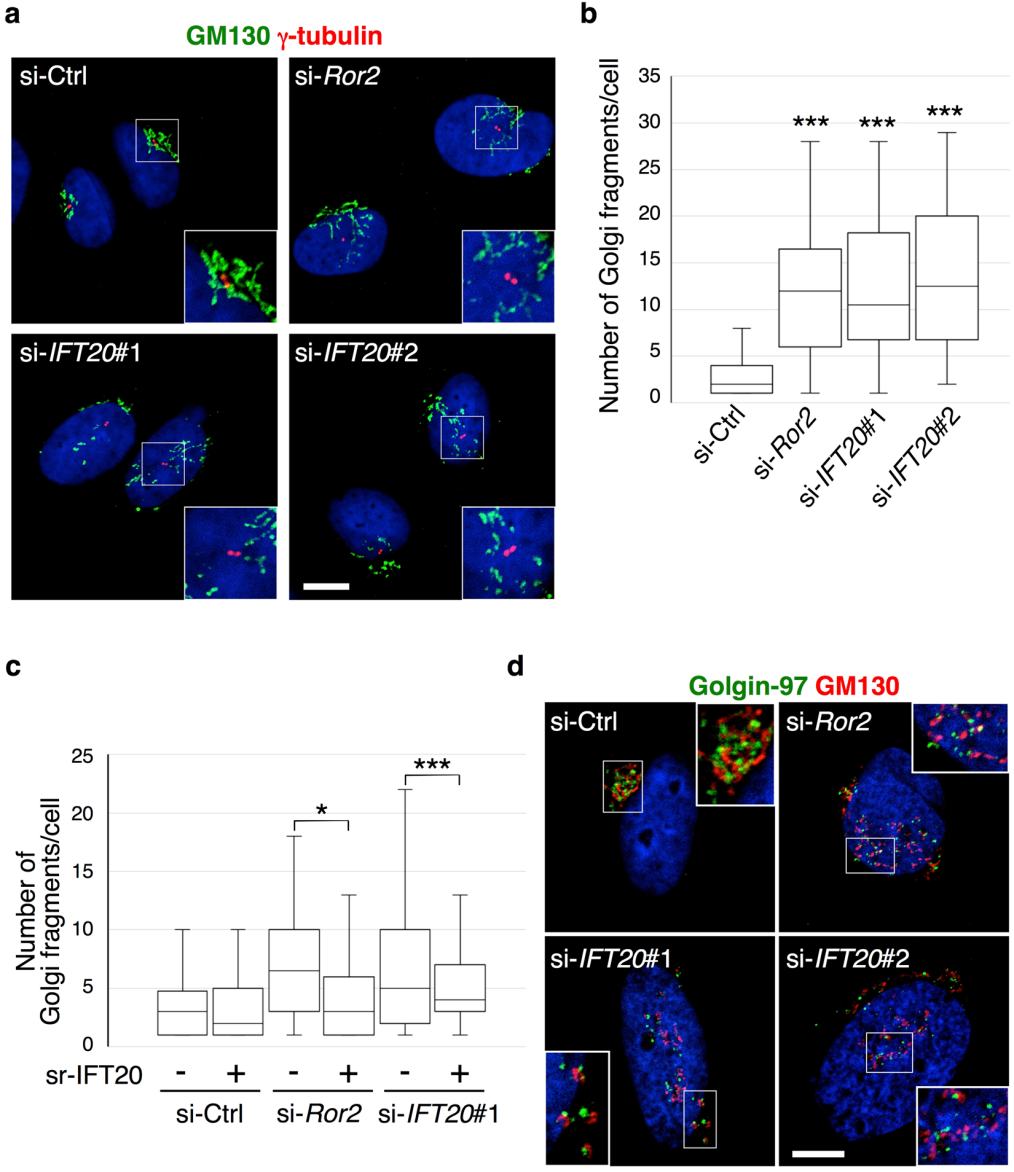
IFT20 is required for assembly of the Golgi apparatus. (**a**) SaOS2 cells transfected with the indicated siRNAs were stained with antibodies against GM130 and γ-tubulin to visualize the *cis*-Golgi and centrosome (centriole pair), respectively, and counterstained with DAPI (blue). Insets show magnified images of boxed regions. Scale bar, 10 µm. (**b**) Number of Golgi fragments per cell was quantified and presented as a box-and-whisker plot. n = 69–74, three independent experiments; ***P < 0.001, *t* test. (**c**) Golgi dispersion in *Ror2-* or *IFT20*-knockdown cells can be suppressed by ectopic expression of IFT20. SaOS2 cells transfected with si-Ctrl, si-*Ror2* or si-*IFT20*#1 were further transfected with *pIRES2-ZsGreen1-sr-IFT20* (+) or *pIRES2-ZsGreen1* (−), as indicated. Number of Golgi fragments in ZsGreen1-positive cells was quantified and presented as a box-and-whisker plot. n = 52–70, three independent experiments; *P < 0.05, ***P < 0.001, *t* test. (**d**) Effects of suppressed expression of *Ror2* or *IFT20* in SaOS2 cells on distribution of the *cis*-Golgi and TGN marker proteins. SaOS2 cells transfected with the indicated siRNAs were stained with antibodies against GM130 and Golgin-97 to visualize the *cis*-Golgi and TGN, respectively, and counterstained with DAPI (blue). Insets show magnified images of boxed regions. Scale bar, 10 µm.

We then sought insight into how siRNA against IFT20 could disrupt the Golgi structure to affect cell polarization. Initially, we found that knocking down not only *IFT20*, but also *Ror2*, induced Golgi dispersion, as tracked through GM130, a Golgi marker (Figs 3a and 4a,b). Ectopic expression of sr-IFT20 restored the effect of siRNA against *IFT20* (Fig. 4c), confirming the specificity of the siRNA targeting. Moreover, ectopic expression of IFT20 also overcame the effect of siRNA against *Ror2* (Fig. 4c). Thus, as cell migration and invasion have been known to require the Golgi to adopt a ribbon-like morphology in promoting polarized secretion to the cell surface^20–22^, the collective findings suggested IFT20 could be regulating Golgi ribbon formation in mediating the ability of Ror2 signaling to promote tumor invasion.

To further pursue this possibility, we next characterized the disassembly of the Golgi ribbon induced by siRNA against either *Ror2* or *IFT20*. We performed double-staining of cells with antibodies against *cis*-Golgi (GM130) and *trans*-Golgi (Golgin-97) markers. In control cells, these markers were closely co-distributed, as would be expected for intact Golgi stacks (Fig. 4d). Notably, these two markers remained closely co-distributed in cells treated with siRNA against either *Ror2* or *IFT20*, despite the overall Golgi ribbon structure having become dispersed (Fig. 4d). Thus, IFT20 likely mediates the Ror2-induced assembly of the Golgi by affecting the assembly of Golgi ribbons, rather than the stacking of Golgi cisternae.

We also examined whether IFT20 regulates the Golgi structure similarly in other tumor cells whose invasiveness might be promoted by the constitutive activation of Ror2 signaling. Examining BT549 (breast cancer) and U2OS (osteosarcoma) cells as examples, which are also non-ciliated (Supplementary Fig. 3a), we first confirmed that siRNA against either *Ror2* or *IFT20* (Supplementary Fig. 3b) also reduced the invasiveness of these tumor cells (Supplementary Fig. 3e). We then found that these siRNA treatments also led to Golgi dispersion (Supplementary Fig. 3c and d). Moreover, targeting specificity of siRNA against *IFT20* was confirmed by the ectopic expression of sr-IFT20, which reverted Golgi dispersion induced by siRNA against *IFT20* (Supplementary Fig. 3b,c and d). We also confirmed that IFT20 mediates the effect of Ror2 signaling in these tumor cells, as the ectopic overexpression of IFT20 reverted the effect of siRNA against *Ror2* (Supplementary Fig. 3b,c and d).

We further considered that Ror2 signaling has been shown to suppress canonical Wnt signaling in these tumor cells. Thus, we wondered whether IFT20 also mediates this process. Wnt signaling enhances TCF/LEF-mediated transcription of its target genes. Examining this activity through a reporter construct (TCF/LEF-driven luciferase reporter), we found that siRNA against *Ror2* inhibited this activity, but siRNA against *IFT20* did not (Supplementary Fig. 3f). Thus, the findings revealed that IFT20 only mediates the ability of the Ror2 signaling to promote Golgi ribbon formation for cell invasiveness, but not the ability of Ror2 signaling to suppress canonical Wnt signaling.

We also noted that IFT20 has been found previously not to regulate the Golgi structure in RPE cells^31^. As these cells are ciliated, we examined whether hMSCs, which are also ciliated cells (see Supplementary Fig. S1), would behave like RPE cells with respect to the regulation of the Golgi by IFT20. We found that knocking down neither *Ror2* nor *IFT20* induced Golgi dispersion in hMSCs (Supplementary Fig. S4). Thus, these observations suggested that, upon the loss of the cilium associated with tumor invasiveness induced by Ror2 signaling, IFT20 in these non-ciliated cells acquires a new function at the Golgi in regulating the structure of this organelle.

**Figure 5:**
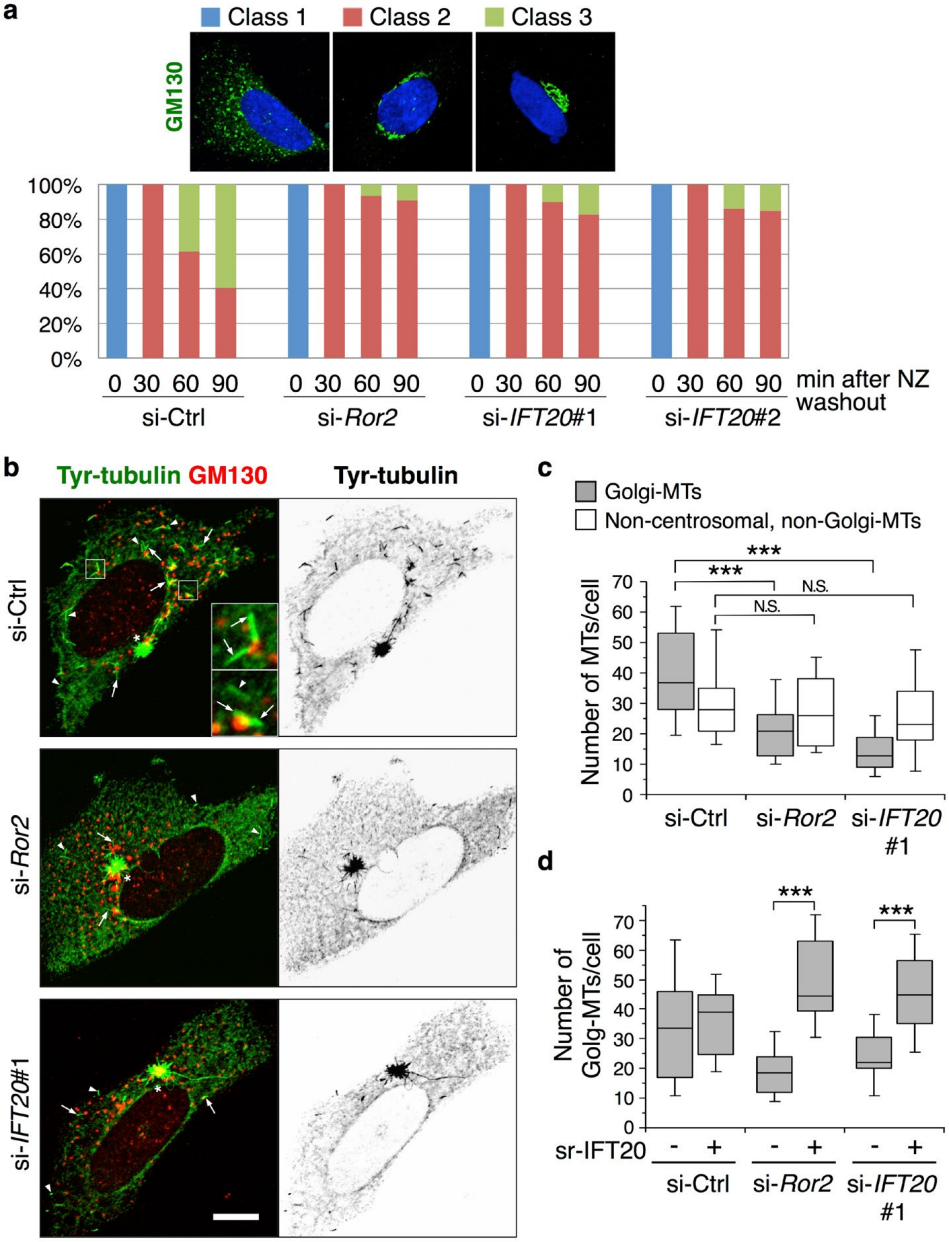
IFT20 is required for MT nucleation at the Golgi. (**a**) Effect of suppressed expression of *Ror2* or *IFT20* on Golgi reassembly during MT repolymerization. SaOS2 cells transfected with the indicated siRNAs were treated with 0.5 µg/ml nocodazole (NZ) for 3 hr. After removal of NZ, cells were incubated for 0, 30, 60, and 90 min at 37 °C. Fixed cells were stained with anti-GM130 antibody (green) and DAPI (blue). Based on the pattern of the Golgi structures, cells were categorized into 3 classes as shown on the top panels: class 1 (cells with dispersed Golgi throughout the cytoplasm), class 2 (cells with dispersed Golgi around the nucleus), and class 3 (cells with normal compact Golgi). The mean percentages of cells in the respective classes were measured. n = 108–200, four independent experiments. (**b**) Effect of *Ror2* or *IFT20* knockdown on MT nucleation. SaOS2 cells transfected with Ctrl, *Ror2* or *IFT20* siRNA were treated with 3 µg/ml NZ for 2 hr. Cells were washed with ice-cold medium to remove NZ followed by 5 min incubation at 25 °C before fixation. Fixed cells were stained with antibodies against GM130 and tyrosinated (Tyr)-tubulin to visualize the *cis*-Golgi and newly nucleated MTs, respectively. Serial optical confocal z sections were obtained and stacked. Insets show magnified images of boxed regions. The arrows and arrowheads indicate Golgi-derived MTs (Golgi-MTs), one end of which is attached to a Golgi fragment, and non-centrosomal, non-Golgi-MTs, respectively. The asterisks indicate the centrosome. Inverted gray scale images of Tyr-tubulin were shown on the right. Scale bar, 10 µm. (**c**) Number of Golgi-MTs and non-centrosomal, non-Golgi-MTs per cell was quantified. Data are presented as a box-and-whisker plot. n = 50–56, three independent experiments; ***P < 0.001, N.S. = not significant, *t* test. (**d**) Reduced nucleation of Golgi-MTs by suppressed expression of *Ror2* or *IFT20* can be rescued by ectopic expression of IFT20. SaOS2 cells transfected with si-Ctrl, si-*Ror2* or si-*IFT20*#1 were further transfected with *pIRES2-ZsGreen1-siRNA-sr-IFT20* (+) or *pIRES2-ZsGreen1* (−), as indicated. MT nucleation of the respective transfected cells was assessed as described in (**c**), and the number of Golgi-MTs in ZsGreen1-positive cells was quantified and presented as a box-and-whisker plot. n = 24–39, three independent experiments; ***P < 0.001, *t* test.

We then sought to further characterize how IFT20 regulates the ribbon structure of the Golgi. The assembly of Golgi stacks into ribbons has been elucidated in recent years to involve two processes, the translocation of Golgi mini-stacks toward the center of the cells, and the lateral linkage of these mini-stacks into ribbons^26, 41^. A key experimental approach that led to this appreciation has been a pharmacologic approach that allows Golgi disassembly and re-assembly to be examined acutely. This involves treating cells with NZ, a microtubule-destabilizing drug that disassembles the Golgi ribbon into ministacks^23, 24^, followed by NZ washout that allows ribbon re-assembly to be observed acutely. Pursuing this approach of studying Golgi assembly, we initially confirmed in control cells the temporal progression from dispersed Golgi mini-stacks (Fig. 5a, class 1), to their translocation around the nucleus (Fig. 5a, class 2), and then the reformation of the Golgi ribbons (Fig. 5a, class 3). We then examined how siRNA against either *Ror2* or *IFT20* affected these processes. Whereas these siRNA treatments had no significant effect on the transition from class 1 to class 2 phenotype, they reduced the progression from class 2 to class 3 phenotype (Fig. 5a). Thus, the results suggested that IFT20 promotes Golgi ribbon formation by affecting the assembly of Golgi mini-stacks to the ribbon structure.

### IFT20 is required for MT nucleation at the *cis*-Golgi

We then noted that Golgi ribbon formation has been elucidated in recent years to involve a MT network that nucleates at the Golgi, in contrast to the traditional mechanism of nucleation at the centrosome^21, 25, 26^. Thus, we next examined the effect of siRNA against either *Ror2* or *IFT20* on the nucleation of Golgi-derived MTs. Upon NZ washout, cells were stained with an anti-tyrosinated (Tyr)-tubulin antibody to detect newly nucleated MTs^42^. In control cells, three types of MT networks could be appreciated: (i) Golgi-derived, (ii) centrosome-derived, (iii) non-Golgi/non-centrosomal (Fig. 5b,c). Reducing the expression of either *Ror2* or *IFT20* inhibited nucleation of the Golgi-derived MTs, but not the other two pools of MTs (Fig. 5b,c). Notably, the reduced nucleation activity of the Golgi-MTs in *Ror2*- and *IFT20*-knockdown cells was also reverted by the ectopic expression of sr-*IFT20* (Fig. 5d). Thus, as in the case for tumor invasion and invadopodia formation, as well as the regulation of Golgi structure, IFT20 also mediates the regulation of Golgi-derived MTs by Ror2 signaling.

**Figure 6:**
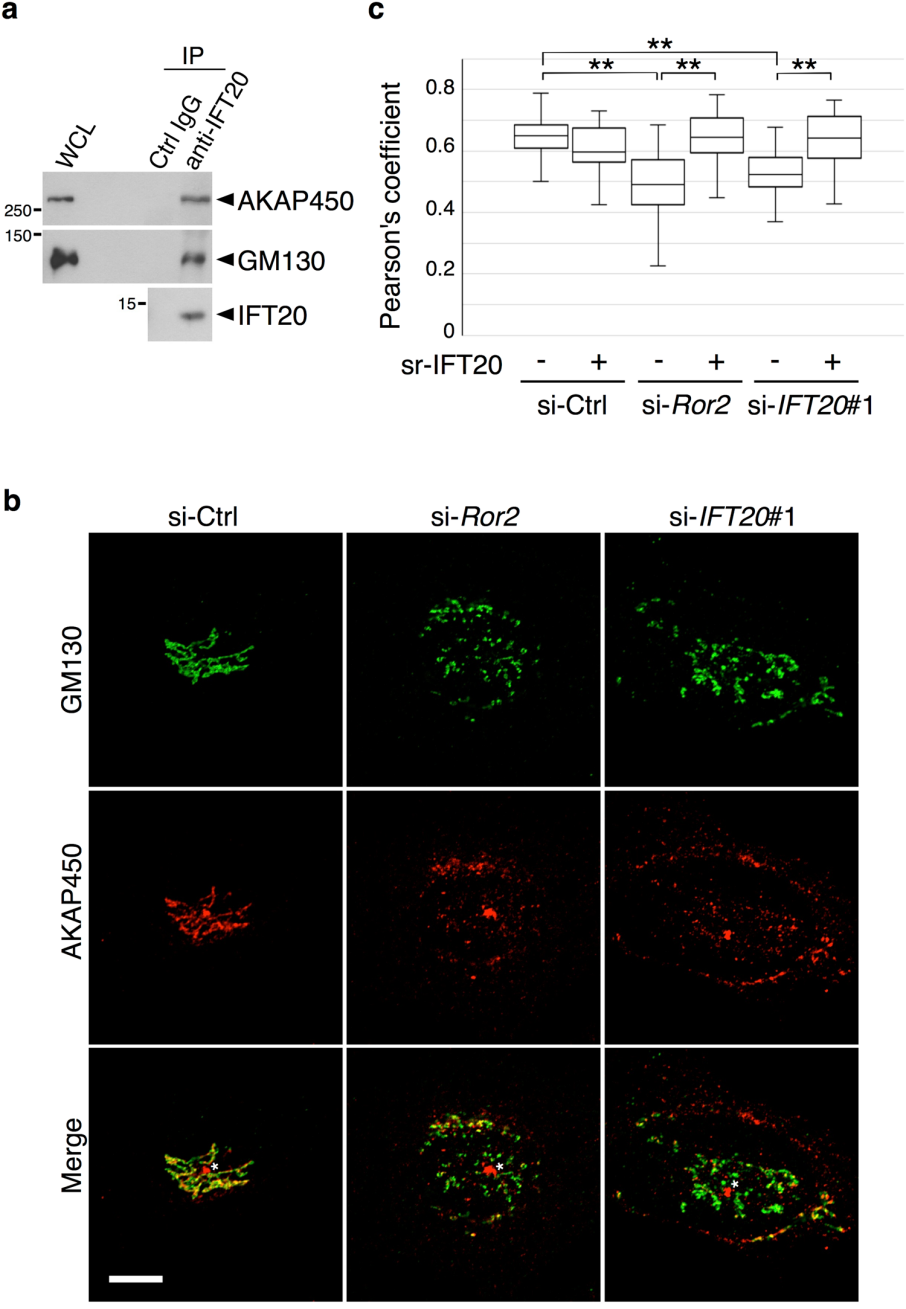
IFT20 is associated with AKAP450 and GM130 and required to maintain expression of AKAP450 at the *cis*-Golgi. (**a**) Co-immunoprecipitation of AKAP450 and GM130 with IFT20 in SaOS2 cells. Whole-cell lysates (WCL) from SaOS2 cells were immunoprecipitated with anti-IFT20 antibody or Ctrl IgG. Immunoprecipitates (IP) and WCL were analyzed by Western blotting with antibodies against the indicated proteins. (**b**) SaOS2 cells transfected with si-Ctrl, si-*Ror2* or si-*IFT20*#1 were stained with antibodies against GM130 and AKAP450. Note that siRNA against *Ror2* or *IFT20* inhibits the colocalization of GM130 and AKAP450. The asterisks indicate AKAP450 at the centrosome. Scale bar, 10 µm. (**c**) SaOS2 cells transfected with si-Ctrl, si-*Ror2* or si-*IFT20*#1 were further transfected with *pIRES2-ZsGreen1-siRNA-sr-IFT20* (+) or *pIRES2-ZsGreen1* (−), as indicated. Colocalization of GM130 and AKAP450 was assessed as described in (**b**) and quantified. Data are presented as a box-and-whisker plot. n = 31–43, three independent experiments; **P < 0.001, *t* test).

We next considered that MT nucleation and/or stabilization at the Golgi could be mediated by molecules localized at both the *cis*- and *trans*-side of the Golgi^25, 27, 43^. As IFT20 resides at the *cis*-side of the Golgi (Supplementary Fig. 2), we next examined whether IFT20 interacts with AKAP450, a molecule that recruits γ-tubulin ring complex (γ-TuRC) to the *cis*-Golgi by forming a complex with GM130 ^27^. Performing a co-immunoprecipitation analysis, we detected both AKAP450 and GM130 interacting with IFT20 (Fig. 6a). We further assessed protein interactions *in situ* by using a proximity ligation assay (PLA)^44^. We found that either anti-AKAP450 or anti-GM130 antibody produced PLA dots when anti-IFT20 antibody was also present, while siRNA against *IFT20* prevented the formation of these PLA dots (Supplementary Fig. 5). We then sought further evidence that IFT20 affects the formation of the AKAP450/GM130 complex at the Golgi. In control cells, AKAP450 was colocalized with GM130 at the Golgi (Fig. 6b,c). However, siRNA against either *Ror2* or *IFT20* reduced this colocalization, which was restored by the ectopic expression of sr-*IFT20* (Fig. 6b,c). In contrast, the siRNA treatment did not affect the localization of AKAP450 at the centrosome (Fig. 6b). Thus, these findings further suggested that IFT20 acts in regulating the nucleation of Golgi-derived MTs by modulating the interaction between AKAP450 and GM130 at the *cis*-Golgi.

Consistent with these findings, treatment of SaOS2 cells with *AKAP450* siRNA, which did not affect the expression level of IFT20 and its colocalization with GM130, also disrupted the Golgi ribbon structure (Supplementary Fig. 6a–d). Moreover, this Golgi dispersion allowed *cis*-Golgi (GM130) and *trans*-Golgi (TGN46) markers to remain closely co-distributed (Supplementary Fig. 6b), suggesting that the assembly of Golgi mini-stacks to ribbons was being affected by siRNA against *AKAP450*, rather than the stacking of Golgi cisternae. Further paralleling the effects of siRNA against *IFT20* and *Ror2*, siRNA against *AKAP450* also inhibited invadopodia formation (Supplementary Fig. 6e). Thus, these additional findings further linked the ability of IFT20 to regulate Golgi ribbon formation with its role in mediating tumor invasiveness and invadopodia formation induced by Ror2 signaling.

### IFT20 promotes anterograde Golgi transport

**Figure 7:**
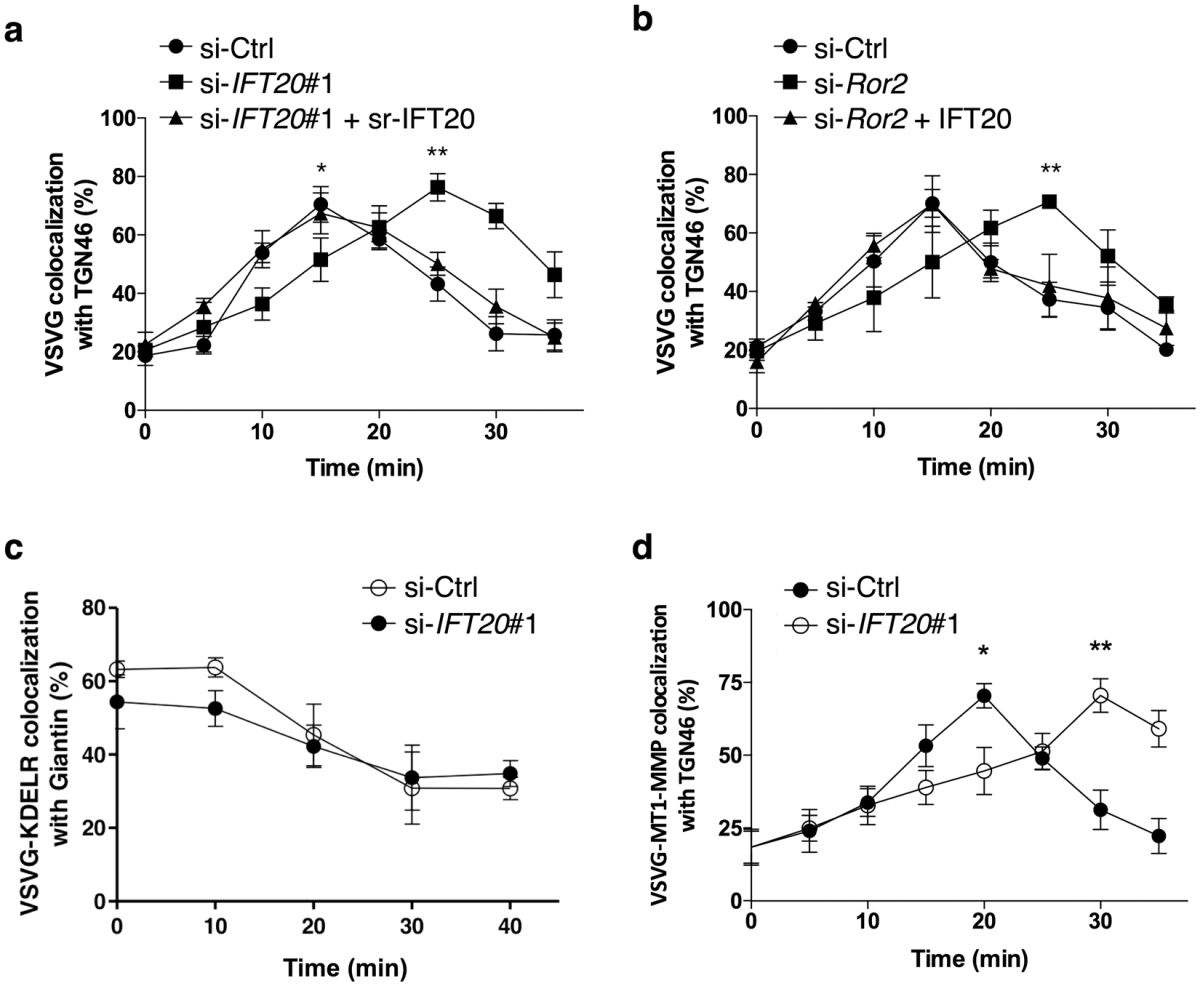
IFT20 is required for efficient anterograde transport through the Golgi. (**a,b**) *cis*-Golgi-to-TGN transport of VSVG is delayed by suppressed expression of *IFT20* or *Ror2*. SaOS2 cells treated with either *IFT20* (**a**) or *Ror2* (**b**) siRNA were further transfected with expression plasmid for VSVG-Myc with or without expression plasmid for sr-*IFT20* (**a**) or *IFT20* (**b**). Cells were incubated at 15 °C to accumulate VSVG-Myc at the pre-Golgi and then shifted to 32 °C. At the indicated time points, colocalization of immunostained VSVG and TGN46, a TGN marker, was examined by confocal microscopy and quantified. Data are expressed as mean ± SEM of three independent experiments. n ≥ 15 in each time point; *p < 0.05; **p < 0.01; Mann Whitney test. Note that delayed transport of VSVG in *IFT20*- (**a**) or *Ror2*-knockdown cells (**b**) is rescued by ectopic expression of sr-*IFT20* or *IFT20*, respectively. (**c**) *IFT20* knockdown fails to affect kinetics of retrograde COPI transport from the Golgi to the ER. SaOS2 cells were transfected with either Ctrl or *IFT20* siRNA along with expression plasmid for VSVG-KDELR-Myc. Cells were incubated at 32 °C and then shifted to 40 °C to allow Golgi-to-ER transport of VSVG-KDELR-Myc. At the indicated time points, colocalization of immunostained VSVG-KDELR-Myc and Giantin, a *cis*-Golgi marker, was examined by confocal microscopy and quantified. Data are expressed as mean ± SEM of three independent experiments. n ≥ 15 in each time point. (**d**) Anterograde transport of VSVG-MT1-MMP through the Golgi is delayed by suppressed expression of *IFT20*. SaOS2 cells were transfected with either Ctrl or *IFT20* siRNA along with expression plasmid for VSVG-MT1-MMP. Anterograde transport of VSVG-MT1-MMP was assessed as described in (**a**). Data are expressed as mean ± SEM of three independent experiments. n ≥ 15 in each time point; *p < 0.05; **p < 0.005; Mann Whitney test.

We then considered that, besides a MT-based mechanism, a transport-based mechanism, which involves anterograde transport through the Golgi, has also been uncovered in recent years to promote Golgi ribbon formation^45–47^. Thus, we also examined whether IFT20 affects this transport. A temperature-sensitive mutant (ts045) of vesicular stomatitis virus glycoprotein (VSVG) has been a model cargo to assess transport through the secretory system, because its transport can be synchronized to allow the quantitative assessment of secretory transport^45–47^. Synchronization is achieved by culturing cells at 40 °C to accumulate VSVG at the endoplasmic reticulum (ER) followed by shifting to 32 °C to allow VSVG to enter the secretory system. Initially, we performed cell surface biotinylation, which revealed that siRNA against *IFT20* inhibited transport of VSVG from the ER to the cell surface (Supplementary Fig. 7a). To further define the segment of the secretory pathway regulated by IFT20, we next examined VSVG transport from the ER to the Golgi. However, siRNA against *IFT20* did not have an appreciable effect on this transport segment (Supplementary Fig. 7b,c). We then examined transport through the Golgi, as previously described^48^. Briefly, this involves shifting cells initially from 40 °C to 15 °C to accumulate VSVG at the *cis*-side of the Golgi, and then further shifting cells to 32 °C to allow VSVG transport through the Golgi^48^. We found that siRNA against either *IFT20* or *Ror2* reduced this transport, which was restored by the ectopic expression of sr-*IFT20* (Fig. 7a,b).

We next considered that enhanced anterograde Golgi transport has been elucidated in recent years to involve the Coat Protein I (COPI) complex generating tubules that connect the Golgi stacks^47, 48^. However, COPI also generates vesicles for retrograde Golgi transport, which can be tracked in cells by following the fate of a retrograde COPI cargo protein, known as VSVG-KDELR^47, 48^. We found that siRNA against *IFT20* did not have an appreciable effect on retrograde COPI transport (Fig. 7c). Thus, IFT20 acts selectively in promoting anterograde, but not retrograde, Golgi transport.

We also examined whether IFT20 regulates the intra-Golgi transport of MT1-MMP, which is a transmembrane protein that has been shown to play a pivotal role in the formation and function of the invadopodia^40, 49, 50^. Previous studies have examined the trafficking of MT1-MMP^40, 49–51^, and found that its cytoplasmic tail (20 amino acids) controls its transport itinerary^52^. Thus, we pursued an established approach of tracking the intra-Golgi transport of any cargo of interest, which involves replacing the cytoplasmic domain of VSVG with the cytoplasmic domain of a different cargo protein of interest^47^. Because the luminal domain of VSVG allows the synchronization of transport, and the cytoplasmic domain of cargoes dictates their transport itinerary, the resulting chimeric VSVG allows the intra-Golgi transport of a particular cargo of interest to be studied^47^. Taking this approach, we found that siRNA against *IFT20* also reduced the intra-Golgi transport of VSVG-MT1-MMP (Fig. 7d).

## Discussion

Invadopodia formation and tumor invasiveness have been found to be promoted by the constitutive activation of Ror2 signaling^14, 15^. In this study, we elucidate how Ror2 signaling exerts these effects through a newly identified mechanism. Initially examining SaOS2 cells as a model tumor, we find that Ror2 signaling promotes the expression of IFT20. We then find that IFT20 mediates the ability of Ror2 to promote the invasiveness of these tumor cells. An initial clue into how IFT20 achieves this role comes from our discovery that IFT20 regulates the ribbon structure of the Golgi. We were then led by previous studies that had found a MT network emanating from Golgi membrane to promote Golgi ribbon formation^26, 53^. Mechanistically, this involves the nucleation of the Golgi MT network through GM130 on Golgi membrane interacting with AKAP450 on MTs^27^. Adding to this mechanistic understanding, we show that IFT20 promotes the GM130-AKAP450 interaction to nucleate Golgi-derived MTs, resulting in Golgi ribbon formation being promoted for tumor invasiveness. The link between the regulation of the Golgi by IFT20 and its role in mediating tumor invasiveness induced by Ror2 signaling is further strengthened by our additional finding that AKAP450 is also needed for the ability of Ror2 signaling to promote tumor invasion and invadopodia formation.

Besides regulating the Golgi structure, we have also uncovered that IFT20 mediates the ability of Ror2 signaling to promote transport through the Golgi complex. In this regard, whereas the role of MTs has dominated the current view of how Golgi ribbon formation occurs, a transport-based mechanism has been uncovered recently. The COPI complex has been known to form transport vesicles in mediating retrograde Golgi transport^47, 48^. Recently, COPI has also been discovered recently to form tubules^47, 48^, which act in two general ways to promote Golgi ribbon formation. One way involves COPI tubules linking Golgi stacks laterally^47^, which is predicted to act in conjunction with the MT-based mechanism to achieve Golgi ribbon formation. A second way involves COPI tubules linking the Golgi stacks longitudinally, which has been suggested to act independently of MTs in promoting a faster rate of anterograde transport through the Golgi^47, 48^. Mechanistically, as we have found that IFT20 promotes anterograde Golgi transport but not retrograde Golgi transport, this finding predicts that COPI is unlikely to be the direct target by which IFT20 enhances anterograde Golgi transport. In considering how IFT20 could act instead, we note that COPI is involved in the formation of Golgi tubules, while the molecular mechanism by which these tubules target an adjacent cisterna to connect the Golgi cisternae remains to be elucidated. Thus, the future identification of the direct target(s) by which IFT20 promotes anterograde Golgi transport will likely come from a better understanding of how COPI tubules connect the Golgi cisternae in forming Golgi stacks.

Our findings also uncover complexities in how IFT20 can act depending on the context. Many cells form a cilium at their surface, which is a MT-based sensory organelle that plays important roles in tissue development and homeostasis^28, 29^. IFT20 is known to reside not only at the cilium, but also at the Golgi^31^. Previous studies on the role of IFT20 at the Golgi suggest that it does not function in regulating Golgi structure in cells that possess primary cilia^31^. Instead, the Golgi-localized IFT20 is suggested to act in transport from the Golgi to the cilium^31–33^. However, many tumor cells lose their cilium during transformation, including breast^54–56^, basal cell^57^, and renal cell carcinoma^58^, as well as osteosarcomas^59^. In these tumors, we have now identified a new function for IFT20, regulating both Golgi structure and transport. This finding is distinct from a non-ciliary role for IFT20 in T cells, which also lack cilia. In these cells, IFT20 at the early endosome has been found to regulate endocytic recycling^34–36^, while we have found that the Golgi pool of IFT20 in non-ciliated tumor cells regulates Golgi structure and transport. We further note that AKAP450 and its binding partner, casein kinase 1δ, in the context of ciliated cells has been suggested previously to regulate the localization and role of IFT20 in promoting transport from the Golgi to the cilium for ciliogenesis^60^. In contrast, we have found in non-ciliated tumor cells that IFT20 regulates the role of AKAP450 in nucleating MTs for Golgi ribbon formation. Thus, these differences add to the growing appreciation for how IFT20 can have different roles depending on whether it is expressed in ciliated versus non-ciliated cells, or whether it resides at the Golgi or at a different compartment in the cell. On a broader level, our findings on how constitutively activated Ror2 signaling induces tumor invasiveness through IFT20 by regulating Golgi structure and transport, have not only shed new insights into how cancer invasiveness can be achieved, but also advance a new understanding for how the function of the Golgi complex can be regulated.

## Methods

### Cells and transfection

SaOS2 and U2OS cells were maintained in DMEM (Nacalai Tesque) containing 10% (v/v) fetal bovine serum (FBS). BT549 cells were maintained in RPMI1640 (Nacalai Tesque) containing 10% (v/v) FBS. Human MSCs were purchased from Lonza (Basel, Switzerland) and maintained in MSCGM medium (Lonza). Cells were transfected with the respective siRNAs and expression plasmids by using Lipofectamine RNAiMAX (Life Technologies) and ViaFect (Promega), respectively, according to the manufacturer’s instructions.

### Antibodies

Rabbit anti-Ror2 ^61^ and anti-IFT20 ^31^ antibodies were prepared as described previously. Sheep anti-TGN46 and rabbit anti-Giantin antibodies have been described previously^62^. Following antibodies were purchased commercially: mouse anti-GM130 (35, BD), anti-Cortactin (4F11, Millipore), anti-γ-tubulin (GTU-88, Sigma), anti-tyrosinated tubulin (TUB01A2, Sigma), anti-AKAP450 (15, BD), anti-GFP (JL-8, Clontech), anti-acetylated tubulin (6-11B-1, Sigma), anti-Myc (9E10, Santa Cruz), anti-Golgin-97 (CDF4, Thermo), and Alexa Fluor 647-conjugated anti-GM130 (5G8, MBL); rabbit anti-IFT20 (13615-1-AP, Proteintech), anti-Arl13B (ab83879, Abcam), anti-GM130 (PM061, MBL), anti-γ-tubulin (T5192, Sigma), anti-Golgin-97 (D8P2K, Cell Signaling Technology), and HRP-conjugated anti-*α*-tubulin (PM054-7, MBL).

### Plasmids and siRNAs

The full length cDNA encoding human *IFT20* was isolated from SaOS2 cells and subcloned into pIRES2-ZsGreen1 vector (Clontech). To construct expression plasmids encoding the sr-*IFT20*, four bases in the targeting sequence within the corresponding human *IFT20* cDNA were altered by PCR-based mutagenesis (GCGTAGAGTACGAAGCTTT) and subcloned into pIRES2-ZsGreen1 vector. Plasmids for VSVG-Myc, VSVG-GFP, and VSVG-KDELR-Myc were gifts from Jennifer Lippincott-Schwartz (National Institutes of Health, Bethesda, MD). The VSVG-MT1-MMP plasmid, encoding the fusion protein consisting of the luminal and transmembrane domains of VSVG (ts045) and the cytoplasmic tail of human MT1-MMP, was constructed by replacing a portion of the VSVG-GFP plasmid encoding both the cytoplasmic tail of VSVG and GFP with the cDNA encoding the cytoplasmic tail of human MT1-MMP. The SuperTopFlash was kindly provided by Randall T. Moon (University of Washington, Seattle, WA). The sequences of the siRNAs used were as follows: si-*IFT20*#1, 5′-GGGUUGAAUAUGAAGCUUUdTdT-3′ (sense) and 5′-AAAGCUUCAUAUUCAACCCdTdT-3′ (anti-sense); si-*IFT20*#2, 5′-GCAAAGACUUUGUGGACAAUU-3′ (sense) and 5′-UUGUCCACAAAGUCUUUGCUU-3′ (anti-sense); si-*AKAP450*, 5′-CUUUGAAGUUAACUAUCAAUU-3′ (sense) and 5′-UUGAUAGUUAACUUCAAAGUU (anti-sense). The sequences of si-*Ror2*, si-*Wnt5a*, and negative control siRNA (si-Ctrl) were described previously^4^.

### RNA isolation and quantitative RT-PCR

Total RNAs were isolated using Isogen (Nippon Gene) and reverse-transcribed using PrimeScript RT reagent kit (TAKARA Bio). Real-time PCR was performed on the LightCycler 480 system (Roche Diagnostics) using LightCycler 480 SYBR Green I Master mix (Roche Diagnostics). The amount of mRNA was normalized relative to that of *18S ribosomal RNA*. The following primers were used: *Ror2*, 5′-CAATTCCACTGGTCATCGCT-3′ (forward) and 5′-TGAGGGGCATTTCCATGTC-3′ (reverse); *Wnt5a*, 5′-TAAGCCCAGGAGTTGCTTTG-3′ (forward) and 5′-GCAGAGAGGCTGTGCTCCTA-3′ (reverse); *IFT20*, 5′-CAGAACTCCTCTAGGGAACCTG-3′ (forward) and 5′-GCTCTATGGTCTGCTGGGTAA-3′ (reverse); *18S ribosomal RNA*, 5′-ATGGCCGTTCTTAGTTGGTG-3′ (forward) and 5′-CGCTGAGCCAGTCAGTGTAG-3′ (reverse).

### DNA microarray analysis

DNA microarray analysis was performed as described previously^63^. Briefly, total RNAs were extracted from SaOS2 cells and used for synthesis of digoxigenin-labeled cRNA probes. Probes were hybridized to Human Genome Survey Microarray v.2.0 (Applied Biosystems). Microarray images were analyzed using Expression Array System Software v1.1.1. (Applied Biosystems). The raw signal intensity was then normalized for aligning the different arrays by global median normalization. The microarray data have been deposited in the NCBI Gene Expression Omnibus (GEO) under accession number GSE76535.

### Immunoprecipitation and Western blotting

Cells were solubilized with lysis buffer [50 mM Tris-HCl (pH7.5), 150 mM NaCl, 5 mM EDTA, 0.5% (v/v) NP-40, 50 mM NaF, 1 mM Na_3_VO_4_, 10 µg/ml leupeptin, 10 µg/ml aprotinin, 0.25 mM pAPMSF] and centrifuged at 12,000 × g for 20 min. For co-immunoprecipitation assay, cells were sonicated briefly in lysis buffer before centrifugation. The lysates were subjected to immunoprecipitation, SDS-PAGE, and Western blot analyses as described^2^.

### Immunofluorescence and Microscopy

Cells were cultured on glass coverslips (Matsunami) pre-coated with 10 µg/ml fibronectin (Sigma) and fixed with 4% (w/v) paraformaldehyde in PBS or BRB80 [80 mM Pipes (pH 6.8), 1 mM MgCl_2_, and 1 mM EGTA] for 10 min at room temperature. Fixed cells were stained with the respective antibodies, phalloidin conjugated with either Alexa Fluor 488 or rhodamine (Invitrogen), along with DAPI (Sigma) as described previously^2, 54^. *In situ* proximity ligation assay (PLA) was performed using Duolink kit (Olink Bioscience) according to the manufacturer’s instructions. After completion of the PLA reaction, samples were refixed with 4% (w/v) paraformaldehyde and incubated with Alexa Fluor-conjugated secondary antibodies (Life Technologies) to detect the individual proteins. Fluorescence images were obtained using a laser scanning confocal imaging system (LSM700, Carl Zeiss) and processed using the ImageJ software. Number of Golgi fragments was quantified by using the ImageJ particle analysis tool. Colocalization was examined using the ImageJ JACoP plugin^64^ or Metamorph (Molecular Devices).

### Polarization of the centrosome

The reorientation of the centrosome during tumor invasion was assessed by 2D invasion assay. The two-well culture insert with 0.5 mm gap between wells (ibidi) was placed on a fibronectin-coated glass-bottom dish. SaOS2 cells transfected with the respective siRNAs were plated onto the culture insert and grown to confluent monolayers. After the inserts were removed, the monolayers were washed with PBS and overlaid with Matrigel (BD) diluted 1:20 in PBS, followed by incubation for 4 hr before addition of growth medium. Cells were then cultured for 24 hr to allow invasion toward the space between the monolayers. After fixation with 4% (w/v) paraformaldehyde, cells were stained with antibody to γ-tubulin to visualize the centrosome, and counterstained with DAPI. The percentages of the edge cells in which the centrosome was within the 120° sector emerging from the center of the nucleus and facing toward the space between the monolayers was measured.

### Transwell invasion and ECM degradation assays

Transwell invasion assay was performed as described previously^4^. In brief, cells were loaded onto the upper well of the Transwell chamber with 8 µm ϕ pore membrane (Coster), precoated with Matrigel on an upper side of the chamber. The lower well was filled with 600 µl of DMEM containing 10% FBS. After incubation for 24 hr, cells invaded to lower surface of the membrane were counted. For ECM degradation assay, glass coverslips were coated with gelatin conjugated with either Alexa Fluor 594 (Invitrogen) (Alexa-gelatin) or fluorescein (Invitrogen) (FL-gelatin) as described^65^. Transfected cells were trypsinized, replated on these glass coverslips, and cultured for 6 hr. After fixation, cells were fixed and stained with phalloidin. Number of invadopodia, identified as F-actin dots in the areas of degraded gelatin, was quantified by using the ImageJ particle analysis tool.

### Transport assays

SaOS2 cells were transfected with the respective siRNAs and then with the respective expression plasmids for VSVG-GFP, VSVG-Myc, VSVG-MT1-MMP, or VSVG-KDELR-Myc with or without expression plasmid for sr-*IFT20* or *IFT20*. To examine the ER-to-cell surface transport, VSVG-GFP-transfected SaOS2 cells were incubated overnight at 40 °C in DMEM containing 1% FBS to accumulate VSVG-GFP at the ER, and then shifted to DMEM containing 1% FBS and 0.1 mg/ml cycloheximide pre-warmed at 32 °C to allow transport through the Golgi. After 30 or 60 min in culture, cell surface proteins were biotinylated with 0.5 mg/ml Sulfo-NHS-ss-biotin (Thermo) in Dulbecco’s PBS (DPBS) for 30 min at 4 °C and quenched with 50 mM NH_4_Cl in DPBS for 10 min at 4 °C. After washing with ice-cold DPBS, cells were solubilized with Triton X-100 lysis buffer [25 mM Tris-HCl (pH7.5), 150 mM NaCl, 5 mM EDTA, 1% (v/v) Triton X-100, 0.4% (w/v) sodium deoxycholate, 10 µg/ml leupeptin, 10 µg/ml aprotinin, 0.25 mM pAPMSF]. Biotinylated proteins were affinity-purified with streptavidin-Sepharose beads and subjected to SDS-PAGE followed Western blot analyses. ER-to-*cis*-Golgi and intra-Golgi transport of VSVG and VSVG-MT1-MMP and retrograde transport of VSVG-KDELR have been described previously^48^.

### MT nucleation assay

Transfected cells were treated with 3 µg/ml nocodazole (NZ) in culture medium for 2 hr. Cells were washed with ice-cold DMEM 5 times on ice to remove NZ and then incubated for 0∼8 min in CO_2_-independent medium (Life Technologies) containing 1% (v/v) FBS at 25 °C. Cells were fixed with 4% (w/v) paraformaldehyde in BRB80 for 5 min at 25 °C, followed by 10 min fixation with methanol at −20 °C. Fixed cells were stained with antibodies against GM130 and tyrosinated (Tyr)-tubulin to visualize the *cis*-Golgi and newly nucleated MTs, respectively. Serial optical confocal z sections spanning the entire cell were obtained and stacked using a maximal intensity projection. Number of Golgi-MTs (non-centrosomal MT that has one end attached to a Golgi fragment) and non-centrosomal, non-Golgi-MT was quantified from the z sections and their stacked images.

### Reporter Assay

Cells were transfected with the respective siRNAs. After two days in culture, cells were further transfected with the SuperTopFlash reporter plasmid together with the internal control plasmid pGL4.74[*hRluc*/TK] (Promega) at the ratio of 100:1 and cultured for one day. Luciferase activities were measured by using Dual-Luciferase Reporter Assay System (Promega) and GloMax 96 Microplate Luminometer (Promega), according to the manufacturer’s instructions.

## Electronic supplementary material

Supplementary Figures 1–7
