# Supplementary material for: Ror2 signaling regulates Golgi structure and transport through IFT20 for tumor invasiveness
Source: Sci Rep. 2017 Jan 26;7:1. doi: 10.1038/s41598-016-0028-x (PMC5428335; doi:10.1038/s41598-016-0028-x)
Supplement: Supplementary file 1 — Supplementary Figures 1–7 [file 41598_2016_28_MOESM1_ESM.pdf]

Supplementary Figures

Ror2 signaling regulates Golgi structure and transport through IFT20 for tumor invasiveness

Michiru Nishita, Seung-Yeol Park, Tadashi Nishio, Koki Kamizaki, ZhiChao Wang, Kota Tamada, Toru Takumi, Ryuju Hashimoto, Hiroki Otani, Gregory J. Pazour, Victor W. Hsu, Yasuhiro Minami

Supplementary Figure 1

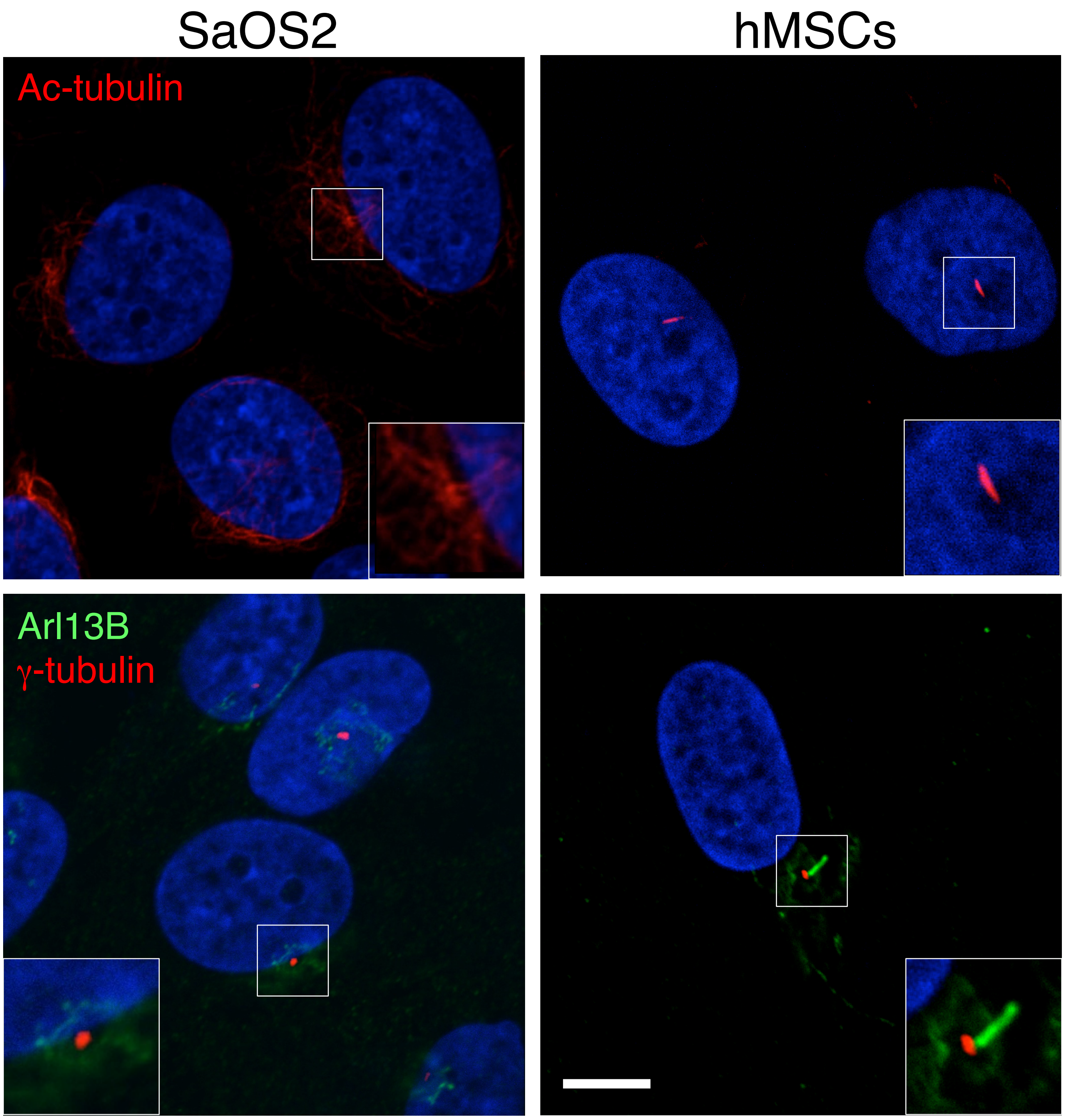

Supplementary Figure 1. SaOS2 cells are non-ciliated. Confluent monolayers of SaOS2 cells or human bone marrow-derived mesenchymal stem cells (hMSCs) were serum starved for 24 hr and fixed. Cells were stained with antibodies against acetylated tubulin (Ac-tubulin) (upper panels) or Arl13B (lower panels) to visualize primary cilia and counterstained with DAPI (blue). Cells were also stained with anti-γ-tubulin antibody to visualize centrosome/basal body (lower panels). Insets show magnified images of boxed regions. Note that Ac-tubulin in hMSCs was imaged with lower detector gain due to its high fluorescence intensity at the primary cilia. Scale bar, 10 μm.

Supplementary Figure 2

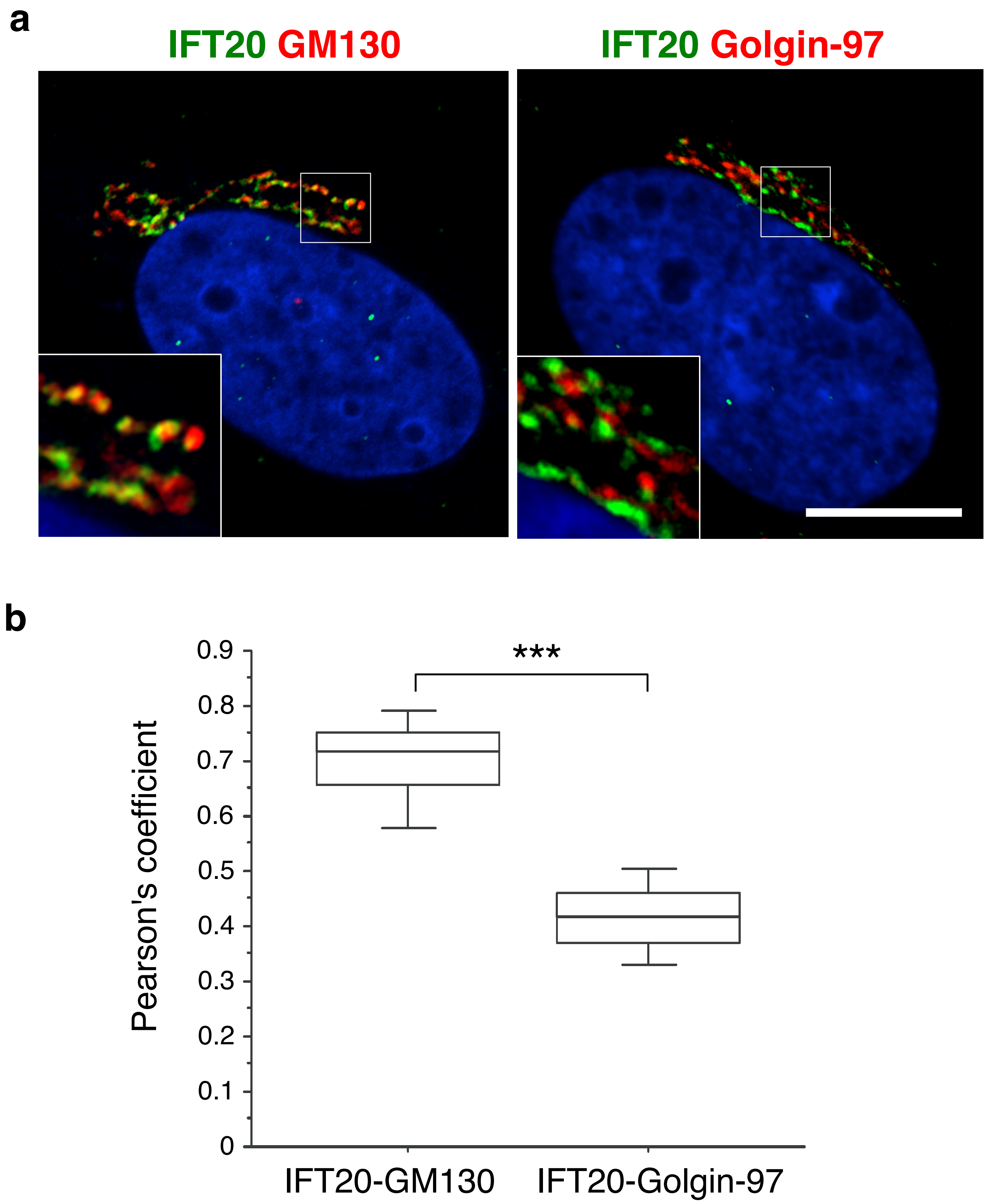

Supplementary Figure 2. Localization of IFT20 at the *cis*-Golgi. (**a**) SaOS2 cells were subjected to immunofluorescence and counterstained with DAPI (blue). IFT20 is colocalized with GM130 (*cis*-Golgi marker), but not Golgin-97 (TGN marker). Insets show magnified images of boxed regions. Scale bar, 10 µm. (**b**) Colocalization of IFT20 with GM130 or Golgin-97 shown in (**a**) was quantified. Data are presented as a box-and-whisker plot. n=29 (IFT20-GM130) and 32 (IFT20-Golgin-97), three independent experiments; $^{***}P<0.001$, *t* test.

Supplementary Figure 3

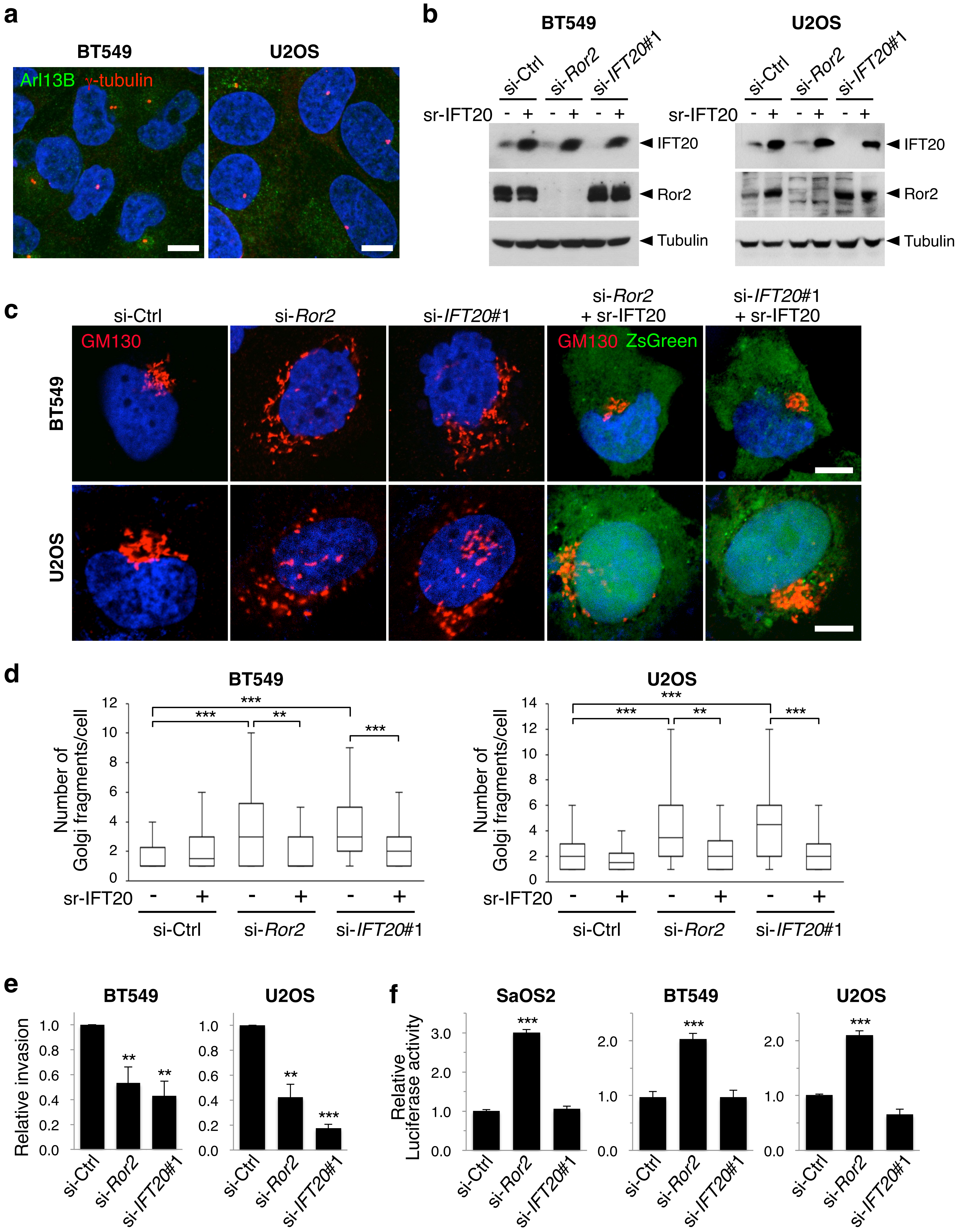

Supplementary Figure 3. Suppressed expression of Ror2 or IFT20 induces Golgi dispersion and inhibits invasive migration of BT549 (breast cancer) and U2OS (osteosarcoma) cells. (**a**) Confluent monolayers of BT549 and U2OS cells were serum starved for 24 hr and fixed. Cells were stained with antibodies against Arl13B and γ-tubulin to visualize primary cilia and centrosome/basal body, respectively, and counterstained with DAPI (blue). Primary cilia were not observed in both BT549 and U2OS cells (n=125 and 107 cells, respectively). Scale bar, 10 μm. (**b**) BT549 and U2OS cells were transfected with the indicated siRNAs and further transfected with *pIRES2-ZsGreen1*-siRNA-resistant (sr)-*IFT20* (+) or *pIRES2-ZsGreen1* (-), as indicated. The respective transfected cells were analyzed by Western blotting with antibodies against the indicated proteins. (**c**) Suppressed expression of *Ror2* or *IFT20* induces Golgi dispersion, which is restored by ectopic expression of sr-IFT20, in BT549 and U2OS cells. Cells transfected with the indicated siRNAs with or without ectopic expression of sr-IFT20, shown by ZsGreen1 expression, were stained with antibodies against GM130 and counterstained with DAPI (blue). Scale bar, 10 μm. (**d**) Cells were transfected with the indicated siRNAs and further transfected with *pIRES2-ZsGreen1-sr-IFT20* (+) or *pIRES2-ZsGreen1* (-), as indicated. The respective transfected cells were analyzed by immunofluorescence as described in (**c**) to quantify the number of Golgi fragments in ZsGreen1-positive cells. Data are presented as a box-and-whisker plot. n=50 for each condition, three independent experiments; $^{**}P<0.01$, $^{***}P<0.001$, *t* test. (**e**) Suppressed expression of *Ror2* or *IFT20* inhibits invasive migration of BT549 and U2OS cells. Cells were transfected with the indicated siRNAs and analyzed by Transwell invasion assay. Cells invaded to the lower surface of the Transwell membranes were counted. Data are expressed as mean ± SD of three (U2OS cells) or four (BT549 cells) independent experiments ($^{**}P<0.01$, $^{***}P<0.001$, *t* test). (**f**) Suppressed expression of *Ror2* enhances the SuperTopFlash reporter activities in SaOS-2, BT549 and U2OS cells. Cells transfected with the indicated siRNAs were subjected to luciferase reporter assays as described in the Methods. Data are representative of three or four independent experiments. The bars represent the mean ± SD of triplicate experiments ($^{**}P<0.01$, $^{***}P<0.001$, *t* test).

Supplementary Figure 4

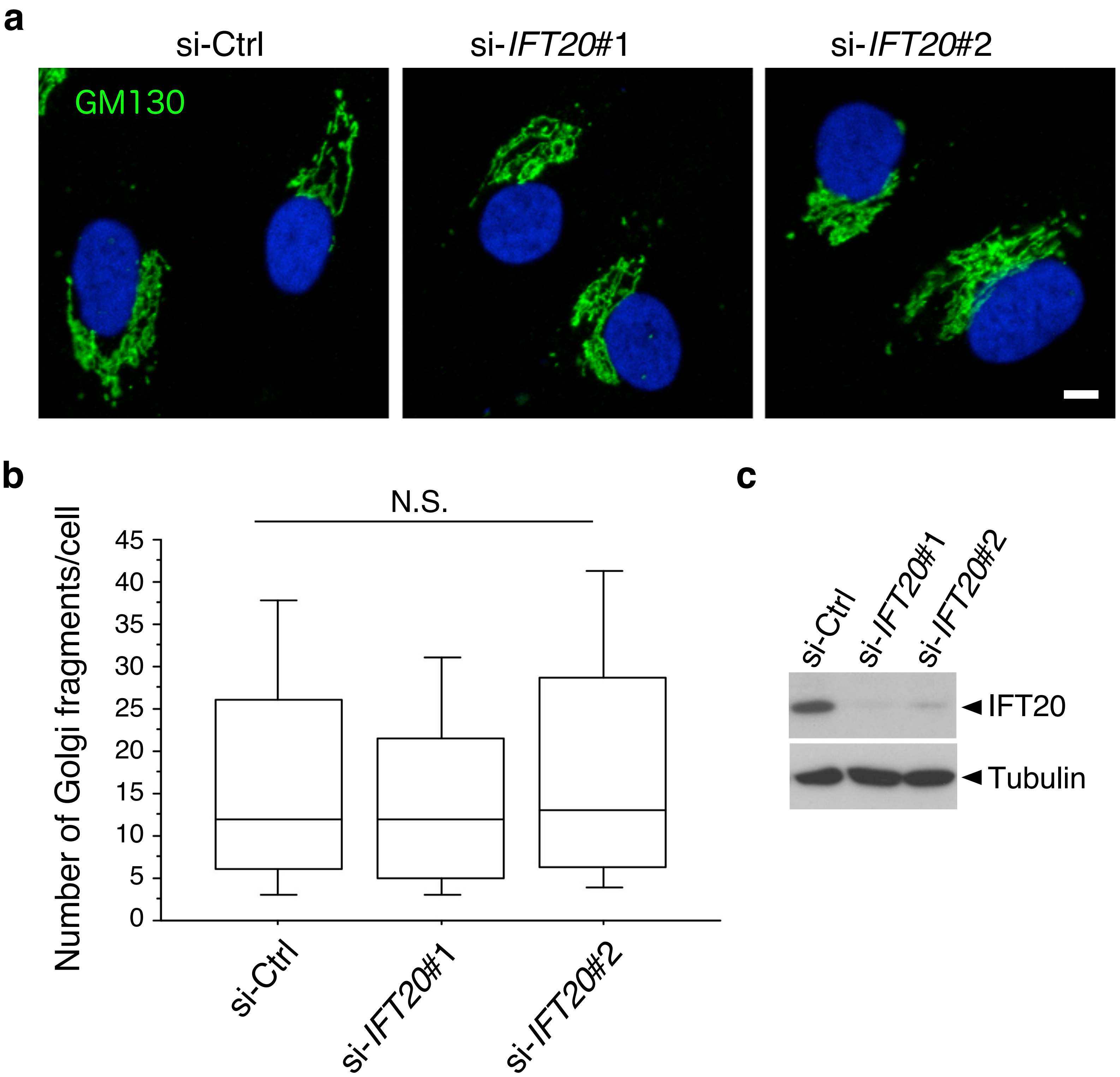

Supplementary Figure 4. Suppressed expression of IFT20 fails to induce Golgi dispersion in human mesenchymal stem cells (hMSCs). (**a**) hMSCs transfected with the indicated siRNAs were stained with antibodies against GM130 and counterstained with DAPI (blue). Scale bar, 10 μm. (**b**) Number of Golgi fragments per cell was quantified. Data are presented as a box-and-whisker plot. n=111-131, three independent experiments; N.S.=not significant, t test. (**c**) Expression levels of IFT20 in siRNA-transfected hMSCs were analyzed by Western blotting.

Supplementary Figure 5

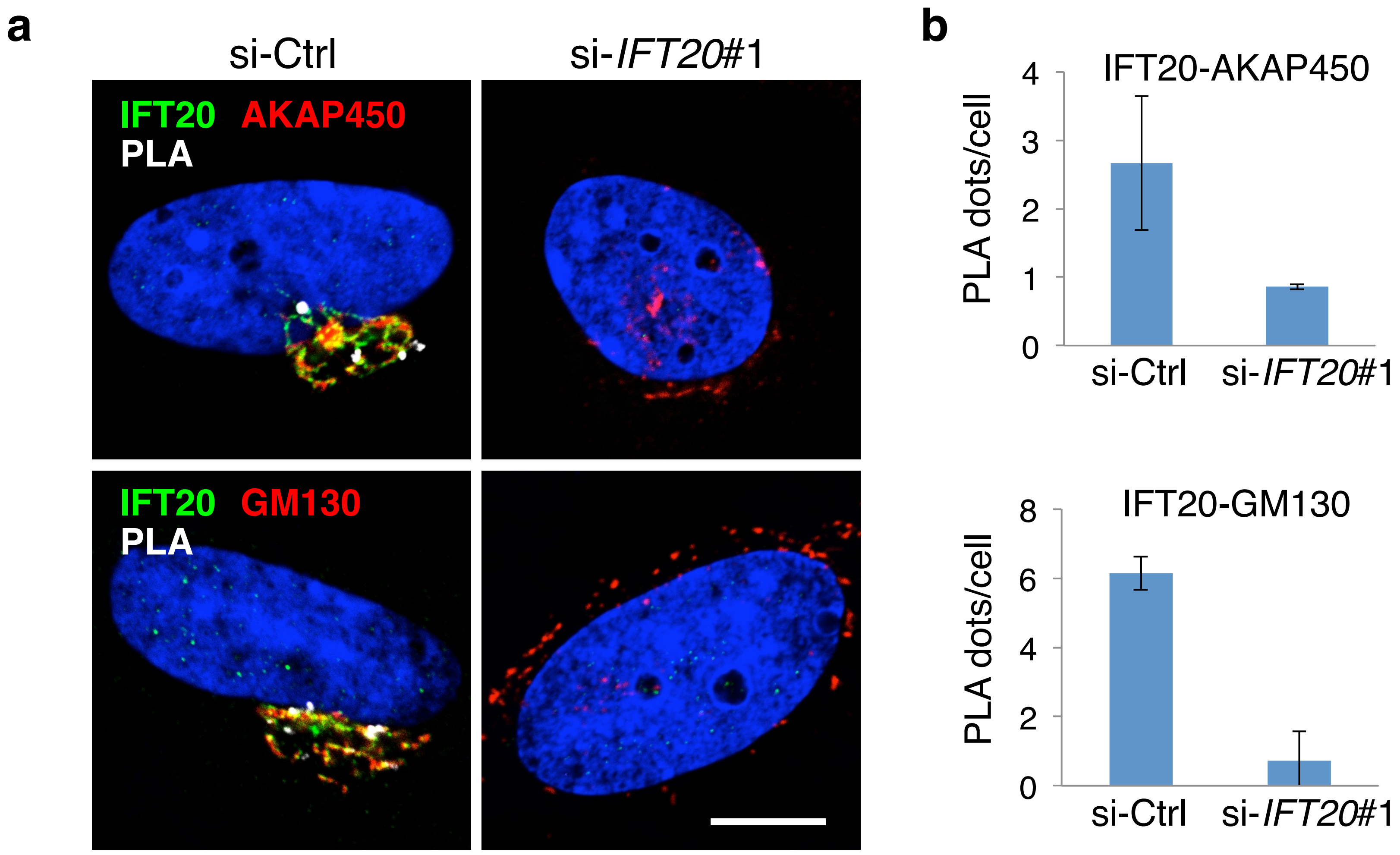

Supplementary Figure 5. IFT20 is localized in close proximity to AKAP450 and GM130 *in situ*. (**a**) SaOS2 cells transfected with either Ctrl or *IFT20* siRNA were analyzed by *in situ* PLA with antibodies against IFT20 and AKAP450 (upper panels) or IFT20 and GM130 (lower panels). The first antibodies used for *in situ* PLA were further detected by immunofluorescence to detect the individual proteins. Cells were counterstained with DAPI (blue). Scale bar, 10 µm. (**b**) The number of PLA dots per cell shown in (**a**) was quantified. Data are expressed as mean ± SD (n=48-131, three independent experiments).

Supplementary Figure 6

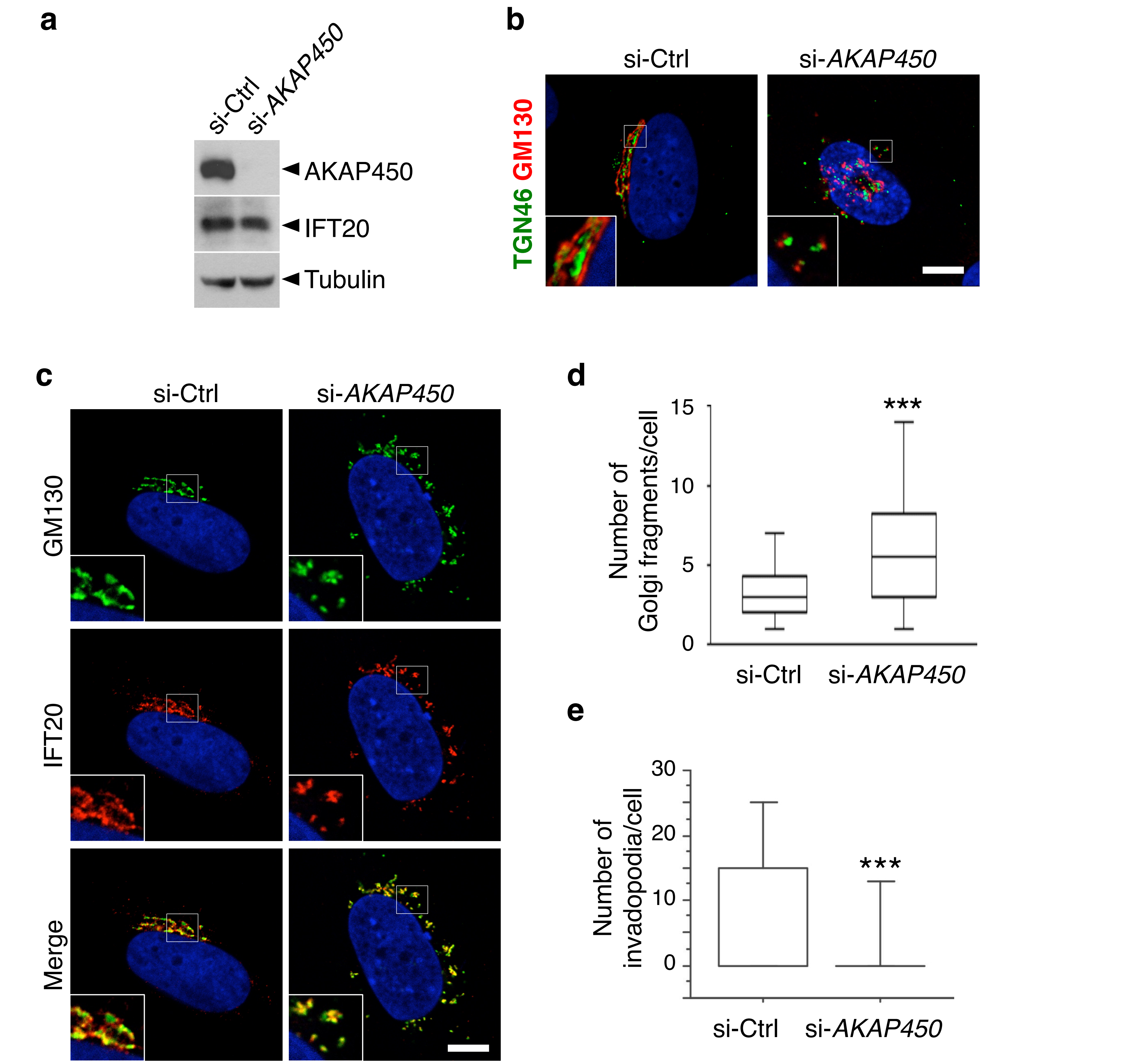

Supplementary Figure 6. Suppressed expression of AKAP450 inhibits invadopodia formation and induces Golgi dispersion in SaOS2 cells. (**a**) Knockdown efficiency of *AKAP450* in SaOS2 cells. SaOS2 cells transfected with *AKAP450* siRNA were subjected to Western blotting with antibodies against the indicated proteins. Note that suppressed expression of AKAP450 fails to affect expression levels of

IFT20. (**b**) Suppressed expression of AKAP450 fails to affect close co-distribution of *cis*-Golgi and TGN. The si-*AKAP450*-treated SaOS2 cells were stained with antibodies against GM130 and TGN46, a TGN marker, to visualize the *cis*-Golgi and TGN, respectively, and counterstained with DAPI (blue). Insets show magnified images of boxed regions. Scale bar, 10 μm. n=50 for each condition, three independent experiments; $^{***}P<0.001$, *t* test. (**c-d**) Suppressed expression of AKAP450 induces Golgi dispersion in SaOS2 cells. The siRNA-treated SaOS2 cells were stained with antibodies against GM130, a *cis*-Golgi marker, and IFT20 and counterstained with DAPI (blue). Insets in (**c**) show magnified images of boxed regions. Scale bar, 10 μm. Note the colocalization of IFT20 and GM130 even in *AKAP450*-depleted cells with dispersed Golgi (**c**). (**d**) Number of Golgi fragments, imaged by anti-GM130 immunostaining, per cell was quantified. Data are presented as a box-and-whisker plot. n=50 for each condition, three independent experiments; $^{***}P<0.001$, *t* test. (**e**) Suppressed expression of AKAP450 inhibits invadopodia formation. The siRNA-treated SaOS2 cells were cultured on glass coverslips pre-coated with FL-gelatin for 6 hr and stained with rhodamine-conjugated phalloidin for F-actin. Number of invadopodia, identified as F-actin dots in the areas of degraded FL-gelatin, per cell was quantified. Data are presented as a box-and-whisker plot. n=123 (si-*Ctrl*) and 187 (si-*AKAP450*), three independent experiments; $^{***}P<0.001$, *t* test.

Supplementary Figure 7

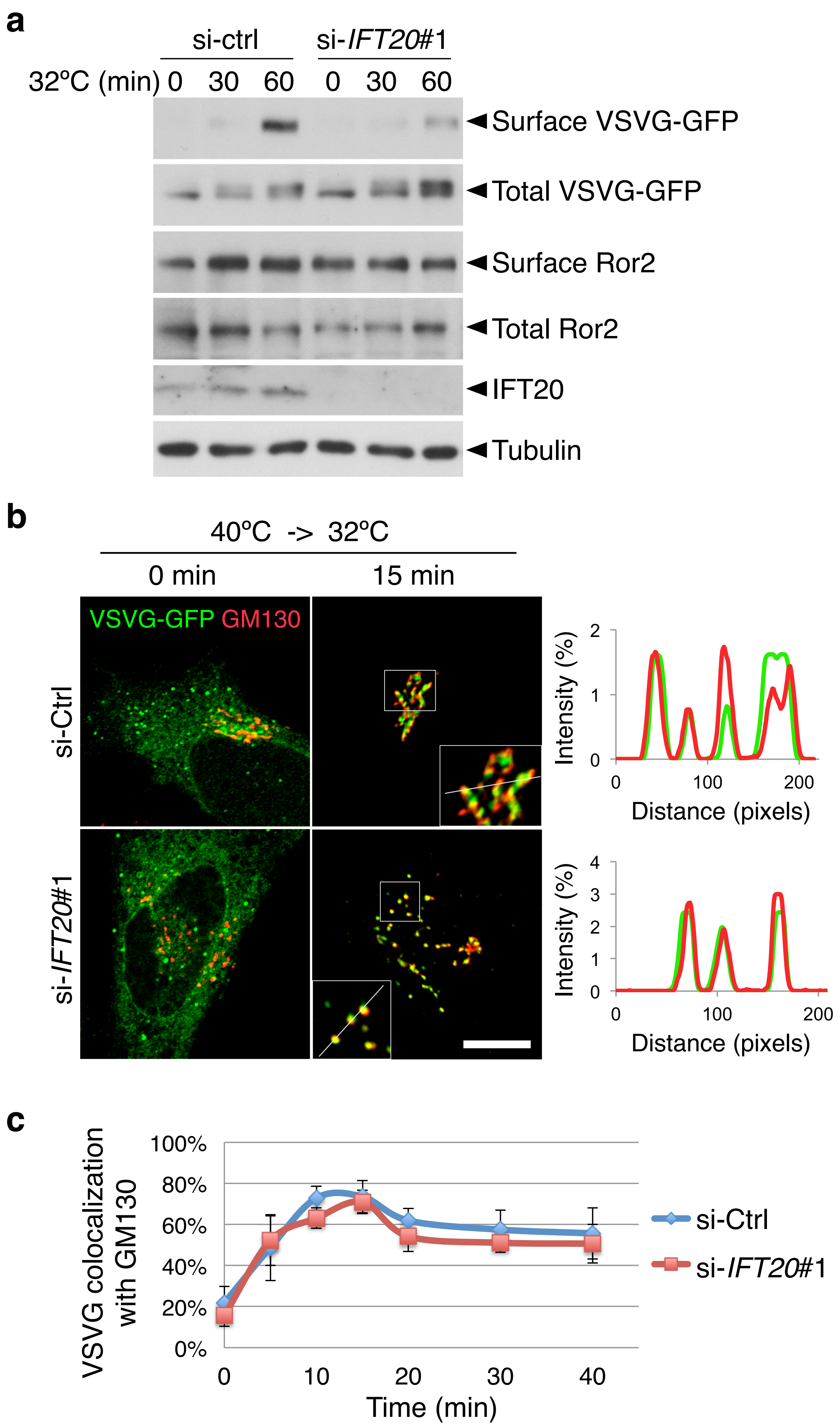

Supplementary Figure 7. Suppressed expression of IFT20 delays ER-to-cell surface transport, but not ER-to-*cis*-Golgi transport of VSVG-GFP. SaOS2 cells were transfected with either Ctrl or *IFT20* siRNA along with expression plasmid for VSVG-GFP. Cells were incubated at 40ºC to accumulate VSVG-GFP at the ER and then shifted to 32ºC to allow its transport through the Golgi. At the indicated time points, VSVG-GFP transported to the cell surface was detected by cell surface biotinylation and subsequent Western blotting (**a**), or cells were fixed and stained with antibodies against GM130 (a *cis*-Golgi marker) (**b, c**). (**b**) Representative merged images for VSVG-GFP (green) and GM130 (red) at 0 min are shown (left panels). Insets show magnified images of boxed regions. Scale bar, 10 µm. Fluorescence intensity profiles of each channel along the white lines are shown (right panels). (**c**) Colocalization of VSVG-GFP and GM130 was quantified at the indicated time points. Data are expressed as mean ± SD (n=8, three independent experiments).
